# Association of Complement and MAPK Activation With SARS-CoV-2–Associated Myocardial Inflammation

**DOI:** 10.1001/jamacardio.2021.5133

**Published:** 2021-12-15

**Authors:** Ludwig T. Weckbach, Lisa Schweizer, Angelina Kraechan, Stephanie Bieber, Hellen Ishikawa-Ankerhold, Jörg Hausleiter, Steffen Massberg, Tobias Straub, Karin Klingel, Ulrich Grabmaier, Maximilian Zwiebel, Matthias Mann, Christian Schulz

**Affiliations:** 1 Medizinische Klinik und Poliklinik I, Ludwig Maximilian University Hospital Munich, Munich, Germany; 2 Institute of Cardiovascular Physiology and Pathophysiology, Biomedical Center, Ludwig Maximilian University Munich, Planegg-Martinsried, Germany.; 3 Munich Heart Alliance, German Centre for Cardiovascular Research, Munich, Germany; 4 Department of Proteomics and Signal Transduction, Max Plank Institute of Biochemistry, Planegg-Martinsried, Germany; 5 Core Facility Bioinformatics, Biomedical Center, Ludwig Maximilian University Munich, Planegg-Martinsried, Germany; 6 Cardiopathology Department, Institute for Pathology and Neuropathology, Tübingen University Hospital, Tübingen, Germany; 7 Novo Nordisk Foundation Center for Protein Research, Faculty of Health Sciences, University of Copenhagen, Copenhagen, Denmark

## Abstract

**Importance:**

Myocardial injury is a common feature of patients with SARS-CoV-2 infection. However, the cardiac inflammatory processes associated with SARS-CoV-2 infection are not completely understood.

**Objective:**

To investigate the inflammatory cardiac phenotype associated with SARS-CoV-2 infection compared with viral myocarditis, immune-mediated myocarditis, and noninflammatory cardiomyopathy by integrating histologic, transcriptomic, and proteomic profiling.

**Design, Setting, and Participants:**

This case series was a cooperative study between the Ludwig Maximilian University Hospital Munich and the Cardiopathology Referral Center at the University of Tübingen in Germany. A cohort of 19 patients with suspected myocarditis was examined; of those, 5 patients were hospitalized with SARS-CoV-2 infection between March and May 2020. Cardiac tissue specimens from those 5 patients were compared with specimens from 5 patients with immune-mediated myocarditis, 4 patients with non–SARS-CoV-2 viral myocarditis, and 5 patients with noninflammatory cardiomyopathy, collected from January to August 2019.

**Exposures:**

Endomyocardial biopsy.

**Main Outcomes and Measures:**

The inflammatory cardiac phenotypes were measured by immunohistologic analysis, RNA exome capture sequencing, and mass spectrometry–based proteomic analysis of endomyocardial biopsy specimens.

**Results:**

Among 19 participants, the median age was 58 years (range, 37-76 years), and 15 individuals (79%) were male. Data on race and ethnicity were not collected. The abundance of CD163+ macrophages was generally higher in the cardiac tissue of patients with myocarditis, whereas lymphocyte counts were lower in the tissue of patients with SARS-CoV-2 infection vs patients with non–SARS-CoV-2 virus-associated and immune-mediated myocarditis. Among those with SARS-CoV-2 infection, components of the complement cascade, including C1q subunits (transcriptomic analysis: 2.5-fold to 3.6-fold increase; proteomic analysis: 2.0-fold to 3.4-fold increase) and serine/cysteine proteinase inhibitor clade G member 1 (transcriptomic analysis: 1.7-fold increase; proteomic analysis: 2.6-fold increase), belonged to the most commonly upregulated transcripts and differentially abundant proteins. In cardiac macrophages, the abundance of C1q was highest in SARS-CoV-2 infection. Assessment of important signaling cascades identified an upregulation of the serine/threonine mitogen-activated protein kinase pathways.

**Conclusions and Relevance:**

This case series found that the cardiac immune signature varied in inflammatory conditions with different etiologic characteristics. Future studies are needed to examine the role of these immune pathways in myocardial inflammation.

## Key Points

**Question:**

What is the cardiac phenotype of patients with SARS-CoV-2 infection compared with viral and immune-mediated myocarditis and noninflammatory cardiomyopathy?

**Findings:**

In this case series of 19 patients undergoing endomyocardial biopsies, cardiac specimens of patients with SARS-CoV-2 infection had a higher abundance of complement-associated factors and serine/threonine protein kinases, with mitogen-activated protein kinase–associated pathways having the highest abundance. Similarities in the cardiac immune signature were found among those with SARS-CoV-2 infection and viral myocarditis.

**Meaning:**

In this study, the exploratory data, which characterized myocardial inflammation by deep phenotyping, have implications for the development of treatment strategies to reduce SARS-CoV-2–mediated tissue injury; these findings require confirmation in a prospective and extended cohort of patients.

## Teaser

This case series uses histologic, transcriptomic, and proteomic profiling to investigate the inflammatory cardiac phenotype among patients with SARS-CoV-2 infection compared with patients with noninflammatory cardiomyopathy and virus-associated and immune-mediated myocarditis.

## Introduction

Inflammatory cardiomyopathy is a common condition characterized by cardiac immune cell infiltration. It can be complicated by heart failure and is associated with adverse outcomes.^1^ Although the condition is predominantly caused by viral infections, a broad variety of factors, including systemic immune disorders and the toxic effects of medications, can produce tissue inflammation and fibrosis leading to impaired cardiac function.^2^ Inflammatory cardiomyopathy has recently been observed in autopsies of patients with SARS-CoV-2 infection.^3^ Furthermore, high cardiac troponin levels indicative of myocardial injury have been frequently reported among hospitalized patients with SARS-CoV-2 infection,^4^ and myocardial inflammation may persist in individuals who recovered from this disease.^5^ Thus, SARS-CoV-2 infection may be associated with inflammatory cardiomyopathy; however, the molecular signatures underlying this condition have remained elusive.

Endomyocardial biopsy (EMB) is an important procedure used to retrieve cardiac specimens and allow diagnostic workup, which includes immunohistochemical and viral genome analyses,^6^ to define the underlying etiologic features of inflammatory cardiomyopathy. However, detailed studies of the molecular mechanisms have been hampered by EMB preservation through formalin fixation and paraffin embedding. Recent advances in molecular biological analysis have enabled researchers to assess coding transcripts of formalin-fixed paraffin-embedded (FFPE) specimens through RNA exome capture sequencing, which applies sequence-specific capture independently of polyadenylated transcripts.^7,8^ Similar progress has been made in the proteomic field by improving the extraction and streamlining the analysis of proteins from FFPE tissue, which is of particular interest because this storage technique allows protein preservation for long periods.^9^ In this regard, mass spectrometry–based proteomic analysis for quantitative profiling of fixed tissue directly from histopathologic slides represents an ideal prerequisite to further process EMB samples after standard diagnostic testing. Using these tools, we performed deep phenotyping of cardiac tissue from patients with SARS-CoV-2 infection and other inflammatory conditions. By combining histologic features, gene expression analysis, and mass spectrometry–based proteomic analysis, we assessed molecular signatures in the hearts of patients with SARS-CoV-2 infection as well as those with viral and immune-mediated myocarditis. The workflow described in this case series may aid in the development of a refined analysis of the immunopathologic characteristics underlying inflammatory cardiomyopathies.

### Methods

This case series was approved by the ethics committee of Ludwig Maximilian University Munich and conducted in accordance with the Declaration of Helsinki.^10^ Participants with SARS-CoV-2 infection were part of the COVID-19 Registry of the Ludwig Maximilian University Hospital Munich (CORKUM; World Health Organization trial identifier: DRKS00021225) and gave written informed consent. Data from the SARS-CoV-2 group were pseudonymized, and data from other groups were deidentified. This study followed the Kempen^11^ reporting guideline for case series.

### Study Population

The study population included 4 groups of patients who received EMBs because of suspected myocarditis. Group 1 comprised 5 patients with SARS-CoV-2 infection, group 2 comprised 4 patients with virus-associated myocarditis (including 2 patients with human herpesvirus 6 infection, 1 patient with Epstein-Barr viral infection, and 1 patient with parvovirus B19 infection), group 3 comprised 5 patients with immune-mediated myocarditis, and group 4 comprised 5 patients with cardiomyopathy without signs of inflammatory origin in the histopathologic workup of their EMB specimens (including 4 patients with hypertensive heart disease and 1 patient with nonischemic dilated cardiomyopathy). Group 4 was considered the noninflammatory control group.

The 5 patients hospitalized with SARS-CoV-2 infection (group 1) were recruited between March and May 2020. Patients in this group had received positive results for SARS-CoV-2 infection on a nasal swab quantitative polymerase chain reaction test; SARS-CoV-2 RNA was not detected in their cardiac tissue. Formalin-fixed paraffin-embedded EMB samples of pseudonymized patients in groups 2 to 4 were collected from the biobank of the University of Tübingen Cardiopathology Department from January to August 2019. All data were then deidentified. Histologic, immunohistologic, and molecular pathologic analyses for the detection of cardiotropic viruses in all samples were conducted during a routine workup at the Institute for Cardiopathology of the University of Tübingen, and further analysis was performed at Ludwig Maximilian University and the Max Planck Institute of Biochemistry. A detailed description of the workflow is available in eFigure 1 in Supplement 1.

### Mass Spectrometry–Based Proteomic Analysis

Formalin-fixed paraffin-embedded material was collected from EMB sections (3 replicates with 5-μm thickness) and processed in a 96-well format using computer-generated randomization (eMethods in Supplement 1). Liquid chromatography-mass spectrometry analysis was performed using an ultrahigh-pressure system (EASY-nLC 1200; ThermoFisher Scientific) coupled to a hybrid trapped ion mobility quadrupole time-of-flight mass spectrometer (timsTOF Pro; Bruker) using a total gradient length of 120 minutes. Mass spectrometry data for each sample were recorded in duplicates using the recently introduced data-independent acquisition mode of the parallel accumulation–serial fragmentation method.^12^ A prefractionated reference library was measured using a top 10 data-dependent mode of the acquisition parallel accumulation–serial fragmentation method.^13^ Data were then processed using Spectronaut software, version 14.9.201124.47784 (Biognosys), using the human (software-integrated) and SARS-CoV-2 (Swiss-Prot; Uniprot Consortium) databases. A detailed description of the mass spectrometry data analysis is provided in the eMethods in Supplement 1.

### RNA Analysis

We conducted RNA isolation of the 19 FFPE EMB samples using a DNA/RNA kit (AllPrep DNA/RNA Mini Kit [80284]; QIAGEN). RNA quality is indicated in eTable 1 in Supplement 1.Sequencing libraries were generated using a library preparation kit (TruSeq RNA Exome; Illumina), and RNA sequencing was performed using a next-generation sequencing system (NovaSeq 6000; Illumina) with a 2 × 75 base pair paired-end run. Sequencing reads were aligned to the human reference genome, version GRCH38.100 (Genome Reference Consortium) using STAR open-source software, version 2.7.3.^14^ Further description of the RNA analysis is provided in the eMethods in Supplement 1.

### Immunohistochemical Analysis

Endomyocardial biopsy specimens were fixed in 4% phosphate-buffered formaldehyde and embedded in paraffin. Four-micrometer–thick tissue sections were stained with hematoxylin-eosin and examined by light microscopy. The presence of vascular thrombi was excluded. For immunohistologic detection of T cells (CD3^15^) and macrophages (CD68^16^), a monoclonal rabbit anti-CD3 antibody (clone SP7, 1:500; Novocastra Laboratories) and a monoclonal mouse antihuman CD68 antibody (clone PG-M1, 1:50; Dako, Agilent Technologies) were used. Immunohistochemical analysis was performed using an automated immunostaining system (BenchMark; Ventana Medical Systems) following the manufacturer's protocol, with a 3,3′-diaminobenzidine detection system (ultraView; Ventana Medical Systems) as substrate. Additional details about immunofluorescence staining and confocal imaging are described in the eMethods in Supplement 1.

### Immunofluorescence Staining and Confocal Imaging

The FFPE EMB samples were cut in serial cross-sections (5-μm thickness), deparaffinized by immersion in xylene and rehydrated in decreasing concentrations of ethanol. Antigen retrieval was achieved using heat and an antigen retrieval buffer (Tris-EDTA Buffer, pH 9.0; Sigma-Aldrich). Samples were washed in phosphate-buffered saline with detergent (0.1% Tween; Sigma-Aldrich) and blocked in phosphate-buffered saline with 10% goat serum. Next, we incubated sections with primary antibodies against CD68 (mouse antihuman CD68, clone 514H12 [MCA1815]; Bio-Rad Laboratories), CD163 (rabbit antihuman CD163, clone EPR19518 [ab182422]; Abcam) or C1q (polyclonal rabbit antihuman C1q [A013602-2]; Agilent Technologies) for 1 hour at room temperature. Samples were again washed in phosphate-buffered saline with detergent (0.1% Tween; Sigma-Aldrich) and incubated with secondary antibodies. For costaining of CD68 and CD163, we used goat antimouse antibodies (Alexa Fluor 555 [A-21424]; Invitrogen) and goat antirabbit antibodies (Alexa Fluor 488 [A11034]; Invitrogen). For costaining of CD68 and C1q, we used goat antimouse antibodies (Alexa Fluor 647 [A21235]; Invitrogen) and biotin goat antirabbit antibodies (biotin [ab207995]; Abcam) with streptavidin (Alexa Fluor 488 [ab272187]; Abcam) as secondary antibodies. Nuclei were stained with nucleic acid stain (Hoechst 33342 [H3570]; Invitrogen). C1q staining was not possible in two samples (1 immune-mediated myocarditis, and 1 noninflammatory control) due to limited sample amounts. Samples were mounted using an antifade mounting medium (S3023; Dako, Agilent Technologies). Images were acquired with a confocal microscope (LSM 880; ZEISS).

### Statistical Analysis

For the RNA analysis, expression values (in transcripts per million) were calculated using RSEM open-source software, version 1.3.3.^17^ An adjusted *P* value (false discovery rate) of less than .10 was set to classify significantly changed expression.

Differential gene expression analysis was performed using DEseq2 software, version 1.28.1 (Bioconductor). Bioinformatics analyses of all mass spectrometry data were performed using the R statistical computing environment, version 4.0.2 (R Foundation for Statistical Computing). In preparation for the analysis, data were filtered stringently for 60% valid intensity values present in each group. For the pairwise comparisons between groups, a 2-sided unpaired *t* test was performed, with q values less than 0.01 and a minimal fold change of 2 considered statistically significant. Biological pathway enrichment analyses (overrepresentation analyses) were conducted in reference to the Reactome database^18^ using the WebGestalt gene set analysis toolkit, version 0.4.4 (Zhang Lab),^19^ and the Benjamini-Hochberg false discovery rate (<0.05).

## Results

Among 19 total participants, the median age was 58 years (range, 37-76 years); 15 participants (79%) were male and 4 (21%) were female (Table). Data on participant race and ethnicity were not collected. The SARS-CoV-2 group comprised 5 individuals (median age, 64 years [range, 41-76 years]; 4 men [80%]) hospitalized with SARS-CoV-2 infection who received EMB because of suspected myocarditis. In general, patients with SARS-CoV-2 infection were moderately ill (3 individuals [60%] admitted to the normal care station) to critically ill (2 individuals [40%] admitted to the intensive care unit), presented with fever (3 individuals [60%]), and had elevated cardiac troponin levels (median, 0.07 ng/mL [IQR, 0.07-0.14 ng/mL]) and signs of nonischemic myocardial tissue injury on magnetic resonance imaging (5 individuals [100%]) (eTable 2 in Supplement 1). We compared this data set to the EMB tissue of 14 patients with other conditions, including 4 patients with non–SARS-CoV-2 viral myocarditis (median age, 62 years [range, 48-75 years]; 4 men [100%]), 5 patients with immune-mediated myocarditis (median age, 53 years [range, 36-58 years]; 3 men [60%]), and 5 patients with noninflammatory cardiomyopathy (median age, 62 years [range, 37-70 years]; 4 men [80%]) (Table). Patients with SARS-CoV-2 infection had preserved left ventricular ejection fraction (median, 53% [IQR, 36%-60%]) compared with patients in other groups (virus-associated myocarditis: median, 30% [IQR, 28%-33%]; immune-mediated myocarditis: median, 25% [IQR, 10%-30%]; noninflammatory control: median, 30% [IQR, 22%-37%]), and 2 patients (40%) with SARS-CoV-2 infection were receiving treatment with immunosuppressant medications (1 was receiving corticoids, and 1 was receiving hydroxychloroquine) at the time of EMB (eTable 3 in Supplement 1).

**Table.** Participant Demographic and Clinical Characteristics

Characteristic	No. (%)				
	All	SARS-CoV-2 group	Noninflammatory control group	Myocarditis	
				Virus-associated group	Immune-mediated group
Total participants, No.	19	5	5	4	5
Age, median (range), y	58 (37-76)	64 (41-76)	62 (37-70)	62 (48-75)	53 (36-58)
Sex					
Female	4 (21)	1 (20)	1 (20)	0	2 (40)
Male	15 (79)	4 (80)	4 (80)	4 (100)	3 (60)
Cardiovascular risk factors					
≤1	15 (79)	4 (80)	3 (60)	4 (100)	4 (80)
>1	4 (21)	1 (20)	2 (40)	0	1 (20)
Coronary artery disease					
Yes	1 (5)	0	0	0	1 (20)
No	16 (84)	5 (100)	4 (80)	3 (75)	4 (80)
NYHA classification of heart failure^a^					
I	0	0	0	0	0
II	4 (21)	0	3 (60)	0	1 (20)
III	10 (53)	3 (60)	2 (40)	3 (75)	2 (40)
IV	4 (21)	2 (40)	0	1 (25)	1 (20)
Arrhythmia^b^					
Yes	2 (11)	0	1 (20)	0	1 (20)
No	17 (89)	5 (100)	4 (80)	4 (100)	4 (80)
Left ventricular ejection fraction, %					
<30	7 (37)	0	2 (40)	2 (50)	3 (60)
30-39	4 (21)	1 (20)	1 (20)	1 (25)	1 (20)
40-55	3 (16)	1 (20)	1 (20)	0	1 (20)
>55	4 (21)	3 (60)	0	1 (25)	0
Intensive care unit^c^					
Yes	4 (21)	2 (40)	0	1 (25)	1 (20)
No	13 (68)	3 (60)	4 (80)	3 (75)	3 (60)
Immunosuppressant medication					
Yes	2 (11)	2 (40)	0	0	0
No	17 (89)	3 (60)	5 (100)	4 (100)	5 (100)

Abbreviation: NYHA, New York Heart Association.

^a^ Class I indicates that ordinary physical activity does not cause the person undue fatigue, dyspnea, or palpitations; class II indicates that ordinary physical activity causes the person fatigue, dyspnea, palpitations, or angina; class III indicates the person is comfortable at rest, and ordinary physical activity causes fatigue, dyspnea, palpitations, or angina; and class IV indicates that symptoms occur at rest.

^b^ Includes sustained and nonsustained ventricular tachycardia.

^c^ At time of biopsy.

**Figure 1.**
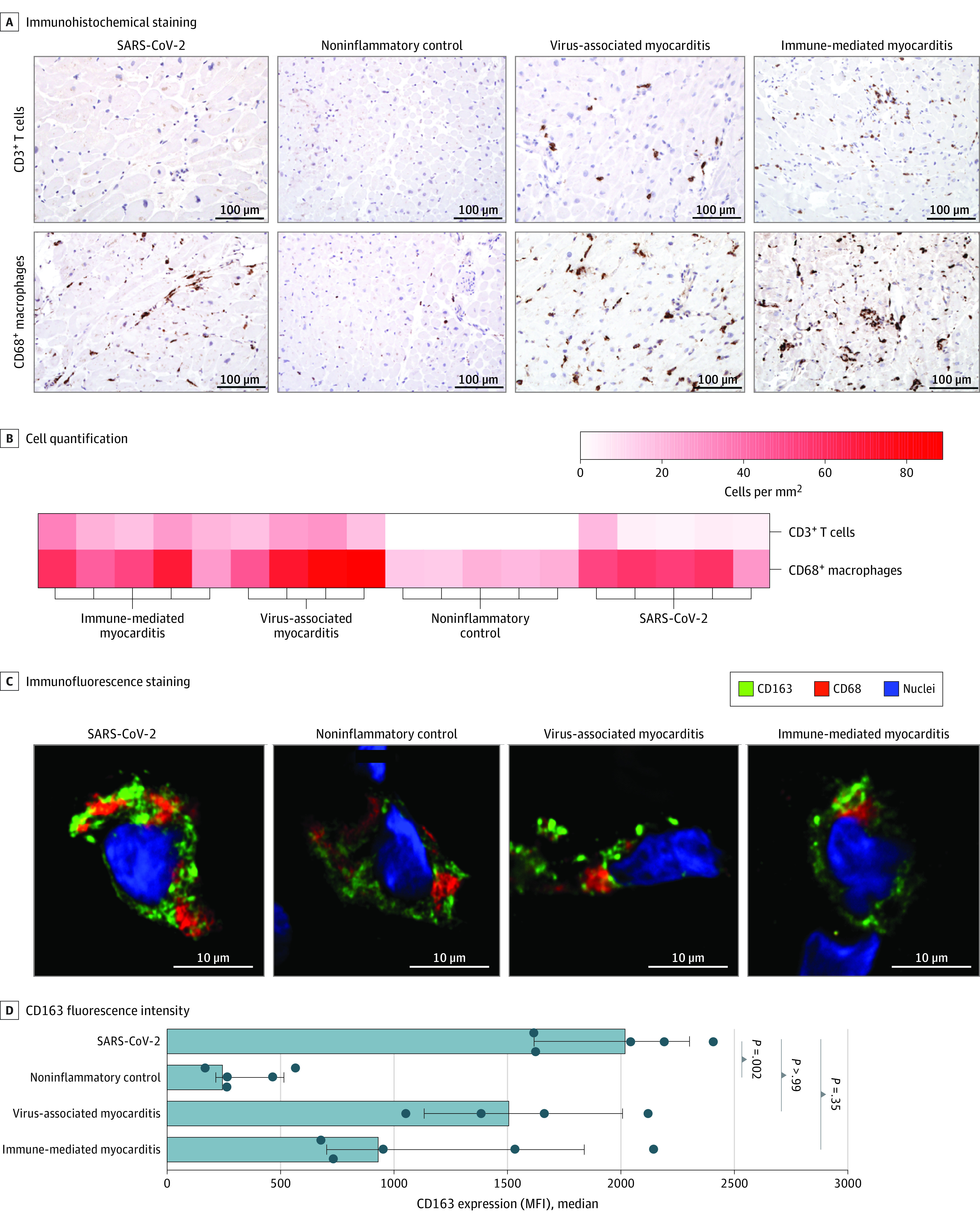
Histologic Characterization of Cardiac Specimens A, Immunohistochemical staining of endomyocardial biopsy (EMB) sections for each group (original magnification, ×200; no hematoxylin-eosin staining was performed). Brown indicates C3+ T cells or CD68+ macrophages. B, CD68+ macrophages and CD3+ T cells per mm^2^ in each EMB sample. C, Confocal images of immunofluorescence expression in cardiac macrophages. Nuclei were stained with DAPI. D, Quantification of CD163 expression (median fluorescence intensity [MFI]) in macrophages for all 4 groups. Error bars represent IQRs. Analysis of variance and Dunn multiple comparison tests were used to determine *P* values; bars to the left of each *P* value indicate the groups being compared. DAPI indicates 4′,6-diamidino-2-phenylindole.

Immunohistochemical analysis of cardiac tissue revealed inflammatory infiltrates consisting of both T cells (CD3) and macrophages (CD68) in patients with immune-mediated and viral myocarditis (Figure 1A and B). Among patients in the noninflammatory control group, T cells were absent, and the abundance of macrophages was low (median, 20 cells per mm^2^ [IQR, 16-22 cells per mm^2^]). Among patients with SARS-CoV-2 infection, the cardiac infiltrate was dominated by macrophages (median, 54 cells per mm^2^ [IQR, 52-57 cells per mm^2^]), and the number of T cells was moderately increased (median, 5 cells per mm^2^ [IQR, 5-6 cell per mm^2^]) (Figure 1B). The abundance of T cells was higher in patients with non–SARS-CoV-2–associated myocarditis (virus-associated myocarditis: median, 23 cells per mm^2^ [IQR, 18-28 cells per mm^2^]; immune-mediated myocarditis: median, 22 cells per mm^2^ [IQR, 21-28 cells per mm^2^]). We next assessed macrophage coexpression of the scavenger receptor CD163, which has recently been found to characterize pulmonary macrophages in patients with SARS-CoV-2 infection.^20^ Although the expression of CD163 increased in patients with all inflammatory conditions compared with those in the noninflammatory control group (Figure 1C), macrophages in the SARS-CoV-2 group had the highest median fluorescence intensity (MFI) values (MFI, 2040 [IQR, 1617-2302]) compared with those in the other groups (virus-associated myocarditis: MFI, 1525 [IQR, 1134-2007]; immune-mediated myocarditis: MFI, 950 [IQR, 706-1836]; noninflammatory control: MFI, 264 [IQR, 214-514]) (Figure 1D). Together, these findings suggest differences in the immune response between the inflammatory conditions examined.

**Figure 2.**
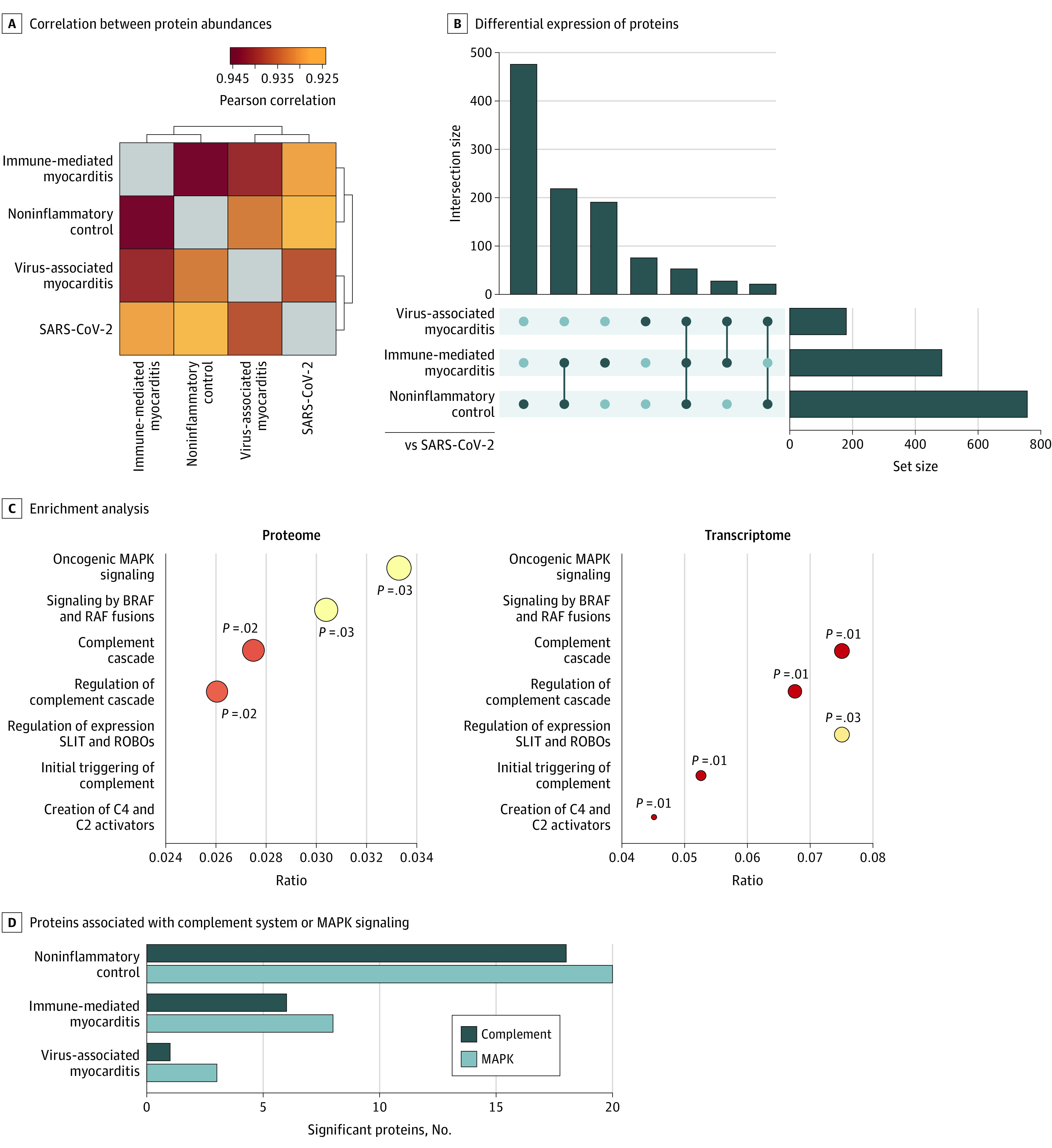
Distinct Molecular Signature in SARS-CoV-2–Associated Inflammatory Cardiomyopathy A, Pearson correlation map of protein abundances indicating similarities and differences of the 4 groups based on proteome. Brackets indicate euclidean hierarchical clustering of samples. B, Shared or unique differential expression of significant proteins (measured using a *t* test, with significance set at q <0.01 and fold change >2) in the specific comparisons between the SARS-COV-2 group (reference) vs the other study groups. The number of significant proteins within each comparison is depicted by the set size, whereas the intersection size represents the quantity of overlap. C, Gene set enrichment analysis of significantly differential proteins and RNA transcripts in the comparison of SARS-CoV-2 vs noninflammatory control samples in reference to the Reactome pathway database^18^ (false discovery rate <0.05). Circle size represents protein or transcript counts for each term, the smallest circle indicating 6, and large circles indicating 23. Circle colors indicate *P* values (range of .01 [red] to .03 [yellow]). D, Count of significant proteins associated with mitogen-activated protein kinase (MAPK) signaling or the complement system in the respective *t* test comparisons of specimens from patients from the SARS-Cov-2 group vs the other study groups. BRAF indicates B-Raf protein; RAF, Raf kinase; ROBO, roundabout receptor; and SLIT, Slit protein.

To explore the molecular signatures of diseased human hearts in an unbiased and comprehensive manner, we characterized the EMB transcriptomes and proteomes (eFigure 1 in Supplement 1). Applying mass spectrometry–based proteomic analysis to the small amount of EMB tissue, we identified more than 3000 proteins in most of the individual cardiac tissue specimens (eFigure 2A in Supplement 1). Overall, the proteomes of EMB tissue from patients with SARS-CoV-2 infection had the highest correlation (*r* = 0.937) with virus-associated myocarditis, whereas proteins of patients in the noninflammatory control group had lower correlation (*r* = 0.928) (Figure 2A). In a principal component analysis, the proteomes of cardiac tissue from patients with SARS-CoV-2 infection substantially differed from those of patients with immune-mediated and viral myocarditis as well as noninflammatory cardiomyopathy, suggesting group-specific alterations in protein abundance (eFigure 2B in Supplement 1).

To characterize the groups in more detail, we examined the differential expression of proteins (eFigure 3 in Supplement 1) and specified the intersection of significantly dysregulated proteins (ie, proteins with q <0.01 and fold change >2) compared with EMB samples from patients with SARS-CoV-2 infection (Figure 2B). Most of the 476 proteins were distinctly differentially regulated compared with those from the noninflammatory control group. In contrast, 53 proteins were commonly dysregulated between the SARS-CoV-2 group and the other groups combined. Among those single molecules, we identified the stromal interaction molecule 1 (STIM1) precursor, which increased in abundance by 5.4-fold among patients with SARS-CoV-2 infection compared with patients with noninflammatory cardiomyopathy (log_2_ fold change, 2.43; −log_10_ [*P* value] = 10.05) (eFigure 3A in Supplement 1). A 5.8-fold and 7.4-fold upregulation was observed in patients with SARS-CoV-2 infection compared with patients with immune-mediated myocarditis (log_2_ fold change, 2.54; −log_10_ [*P* value] = 8.66) (eFigure 3C in Supplement 1, right panel) and viral myocarditis (log_2_ fold change, 2.89; −log_10_ [*P* value] = 4.97) (eFigure 3B in Supplement 1, right panel), respectively. In addition to information on single protein significance, our data set also reached molecular depth to identify the SARS-CoV-2 entry receptor, angiotensin-converting enzyme 2,^21^ in EMB specimens on both the RNA and protein levels (eFigure 4 in Supplement 1). Focusing on shared characteristics between cardiac inflammatory conditions, we next identified 103 proteins that were differentially regulated in patients with SARS-CoV-2 infection, viral myocarditis, and immune-mediated myocarditis compared with patients with noninflammatory cardiomyopathy (eFigure 5A in Supplement 1). Respective upregulated proteins, among other pathways, were associated with immune functions, such as toll-like receptor signaling (eFigure 5B in Supplement 1). These proteins included kinase RPS6KA3 (2.1-fold to 2.7-fold overexpression) and phosphatase subunit PPP2CB (5.3-fold to 7.2-fold overexpression).

To characterize the SARS-CoV-2–associated disease phenotype in biological depth, we performed a pathway enrichment analysis on the proteomic and transcriptomic levels in reference to the noninflammatory control group (Figure 2C). Overall, we identified 2 major themes: (1) serine/threonine kinase signaling, specifically mitogen-activated protein kinase (MAPK) and B-Raf pathways, and (2) complement cascade. Notably, both pathways were also prominent in the distinct protein significance between specimens from the SARS-CoV-2 group and the noninflammatory control group (eFigure 6 in Supplement 1). To specifically address the differences in complement and MAPK pathways between SARS-CoV-2 infection and the other conditions, we quantified the differentially abundant proteins underlying the respective pathways. Although the highest number of significant proteins was found in the comparison between the SARS-CoV-2 group and the noninflammatory control group (20 for MAPK and 18 for complement), complement and MAPK pathway–associated differences were also present in comparison with both immune-mediated myocarditis (8 for MAPK and 6 for complement) and virus-associated myocarditis (3 for MAPK and 1 for complement), albeit to a small extent (Figure 2D).

**Figure 3.**
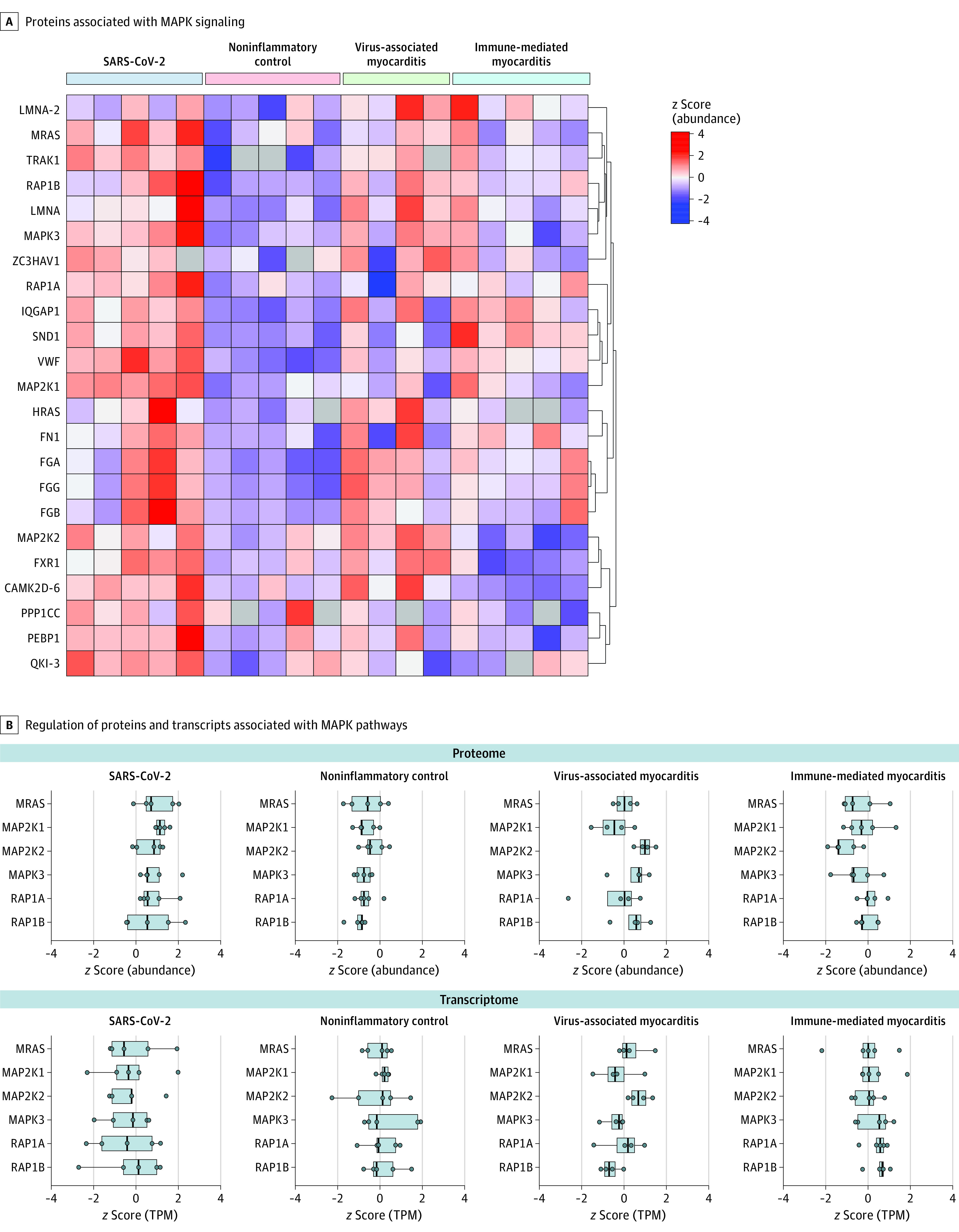
Upregulation of MAPK Pathways in SARS-CoV-2 Infection A, *z* Score normalized abundances of proteins with significant association to MAPK signaling. Row clustering (indicated by brackets) was determined by euclidean distance. B, Points indicate the distribution of *z* scored abundances or TPM values within each group of examples shown in A; boxes represent median values (vertical line) and IQRs. TPM indicates transcripts per million. The subchain expansions are given in the eAppendix in Supplement 1.

We next investigated the major dysregulated themes (MAPK and B-Raf pathways and complement cascade) in more detail. The components of the MAPK pathway, such as MAP2K1 through MAPK3, as well as the Ras-related proteins, RAP1A, RAP1B, and M-Ras, had a median 2.6-fold (IQR, 2.0-fold to 15.5-fold) upregulation among patients with SARS-CoV-2 infection compared with patients with noninflammatory cardiomyopathy (Figure 3). The subchain expansions for Figure 3 are given in the eAppendix in Supplement 1. The EMB specimens of patients with virus-associated myocarditis displayed a similar proteomic profile for a subset of these components (eg, range of fold changes, 2.0-9.5) (Figure 3A). Notably, differential regulation of the MAPK pathways was detected only on the protein level, whereas changes in the transcriptome were minimal (ie, no significant [q <0.01 and fold change >2] differential regulation) (Figure 3B, lower panel; eFigure 7 in Supplement 1, upper panel).

**Figure 4.**
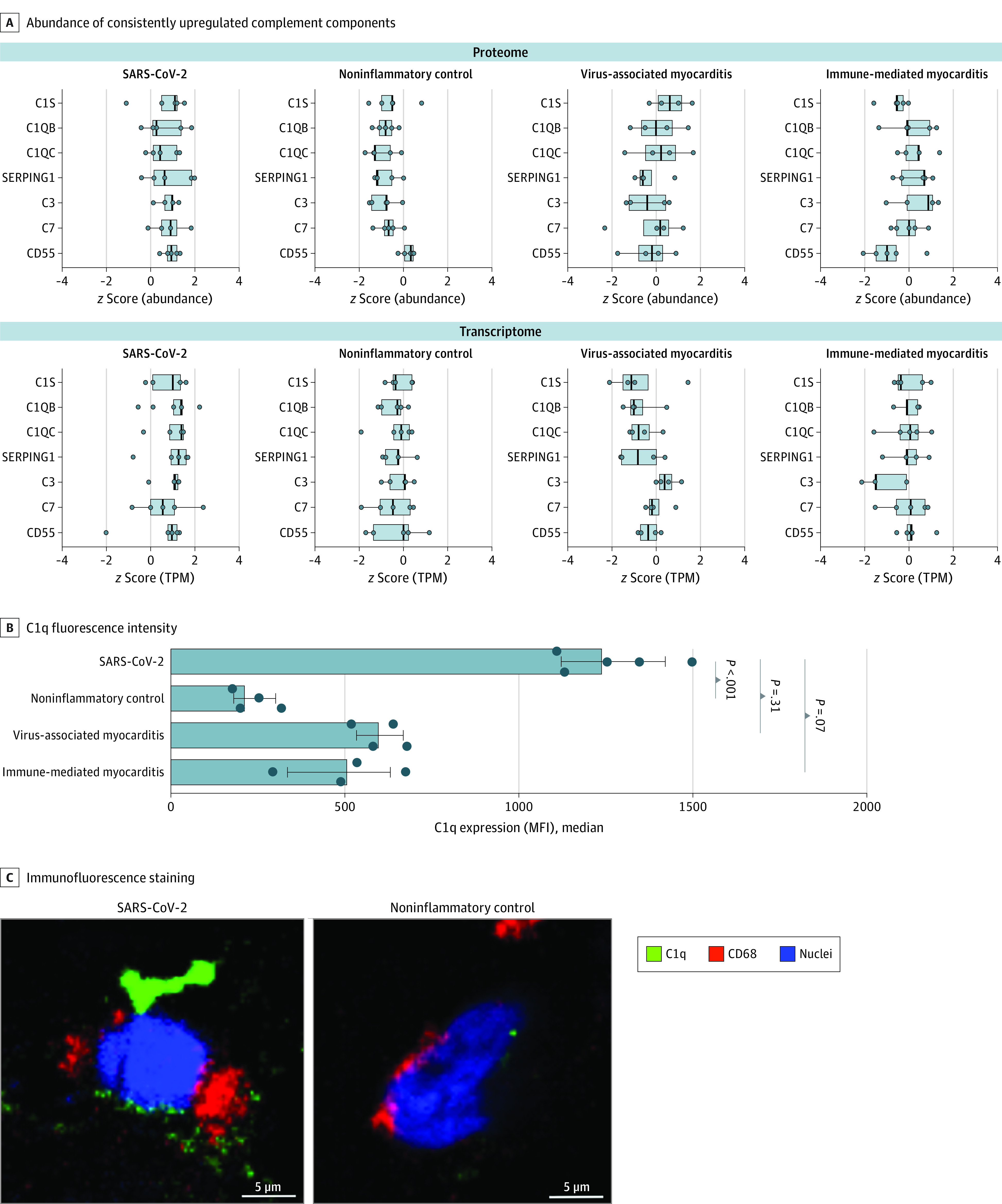
Upregulation of the Complement Cascade in SARS-CoV-2 Infection A, *z* Score–normalized abundances or TPM values of complement components that were consistently upregulated in proteome and transcriptome analysis. TPM indicates transcripts per million. B, Quantification of C1q expression (median fluorescence intensity [MFI]) in macrophages for all 4 groups. C1q staining was not possible in 2 samples (1 immune-mediated myocarditis, and 1 noninflammatory control) due to limited sample amounts. Each dot shows the C1q MFI of 1 patient. Whiskers represent IQRs. Analysis of variance and Dunn multiple comparison tests were used to determine *P* values; bars to the left of each *P* value indicate the groups being compared. C, Representative confocal images of C1q, CD68, and 4′,6-diamidino-2-phenylindole (DAPI, nuclear staining) immunofluorescence in cardiac macrophages from patients with SARS-CoV-2 infection vs noninflammatory cardiomyopathy. SERPING 1 indicates serine/cysteine proteinase inhibitor clade G member 1.

Components of the complement cascade were distinctly upregulated in SARS-CoV-2–associated myocardial inflammation on both the proteomic and transcriptomic levels (Figure 2C). Although RNA transcripts were substantially upregulated in the cohort of patients with SARS-CoV-2 vs noninflammatory cardiomyopathy (range of fold changes, 1.5-4.5), intermediately upregulated abundance stages of complement proteins were also observed in comparison with virus-associated myocarditis (range of fold changes, 1.0-3.2) and immune-mediated myocarditis (range of fold changes, 1.1-5.4) (eFigure 8 in Supplement 1). Among all dysregulated proteins of the complement system, C1qB, C1qC, C3 and C7, in addition to the regulatory proteins, serine/cysteine proteinase inhibitor clade G member 1 (SERPING1) and CD55, were jointly elevated on the proteomic (range of fold changes, 2.0-3.4) and transcriptomic (range of fold changes, 1/5-3.6) levels compared with the noninflammatory stages. Normalized relative abundances revealed upregulation of the complement system in the cardiac tissue of patients with SARS-CoV-2 infection in direct comparison with patients with the other conditions (Figure 4A; eFigure 8 in Supplement 1).

Because C1q can be produced by macrophages,^22^ we specifically addressed the abundance of this complement protein in cardiac macrophages. We identified the presence of C1q in CD68+ macrophages in the immunofluorescence analysis of EMB sections (Figure 4B). Increased expression of this protein was further supported by standardized quantification of C1q median fluorescence intensity values. Macrophage C1q median fluorescence intensity values increased in patients with inflammatory conditions and were highest in patients with SARS-CoV-2 infection (MFI, 1255 [IQR, 1123-1423]) compared with patients with noninflammatory cardiomyopathy (MFI, 225 [IQR, 183-302]), virus-associated myocarditis (MFI, 610 [IQR, 534-669]), and immune-mediated myocarditis (MFI, 504 [IQR, 335-631]). Thus, cardiac macrophages were associated with the increased abundance of complement protein C1q in response to SARS-CoV-2–associated myocardial inflammation.

## Discussion

This case series used RNA exome capture sequencing and mass spectrometry–based proteomic analysis of FFPE cardiac specimens to extend the depth of EMB specimen analysis and assess the biological pathways of cardiac inflammation associated with SARS-CoV-2 infection compared with viral and immune-mediated myocarditis as well as noninflammatory cardiomyopathy. In cardiac tissues of patients with SARS-CoV-2 infection who had suspected myocarditis, the serine/threonine protein kinase pathways associated with MAPK and B-Raf signaling were substantially upregulated on the protein level. The MAPK pathway plays an important role in stress response and immunity.^23^ Signals from pathogen-associated molecular patterns (including viral infections) and other extracellular cues are transmitted into the cell and amplified by MAPK and have been implicated in the pathophysiologic mechanisms of various inflammatory diseases, including cardiac injury. Inhibition of MAPK-associated pathways has been associated with reductions in fibrosis and improvements in heart function in animal models of myocardial injury, offering therapeutic approaches to cardiac inflammation.^24,25^

In addition to distinct dysregulated pathways in SARS-CoV-2 infection, we also identified single molecules that could be of potential interest in cardiac inflammation. For example, we found increased abundance of the STIM1 protein, which has been associated with aberrant Ca^2+^ handling and cardiomyopathy.^26^

Our deep molecular profiling suggested that the complement cascade had an important role in SARS-CoV-2–associated myocardial inflammation. Complement-mediated hyperinflammation is associated with organ injury and could provide targets for new therapeutic strategies.^27^ As a consequence, several clinical trials examining antibodies directed against complement factors (eg, the anti-C5 antibody, eculizumab) are ongoing.^28^ The data provided in this case series add to the findings of a recent study of lung epithelial cells in patients with SARS-CoV-2 infection, in which the complement system was the most highly induced pathway.^29^ In the blood of patients with SARS-CoV-2 infection, increases in SERPING1 and complement component C4A were associated with RNAemia and mortality.^30^ In our study, the components C1q, C3, and C7 of the complement cascade had similar increases in abundance in patients with SARS-CoV-2 infection. We also observed upregulation of the C1 inhibitor, SERPING1, and CD55 (also known as complement decay-accelerating factor), which are considered negative regulators of the complement system.^31,32^ Single-nucleotide variants producing decreased expression of CD55 were associated with an increased risk of adverse clinical outcomes associated with SARS-CoV-2 infection.^33^

Complement activation has also recently been identified in other nonpulmonary organs, such as the liver and kidneys.^29,34^ Innate immune cells are important producers of complement factors.^35^ Notably, increased abundance of complement factors in tissue macrophages may shape their polarization and functions and may be associated with altered immune responses to cardiac injury.^36^ In addition to C1q, cardiac macrophages upregulate CD163, which is generally known as a scavenger receptor and innate immune sensor.^37^ Expression of CD163 in CD68+ cardiac macrophages supports the notion that these are resident immune cells.^38^ Upregulation of CD163 is considered a marker of macrophage activation, and increased expression has been identified in lung macrophages of individuals with SARS-CoV-2 infection.^20^ Consistent with this increased expression, the macrophage inflammatory response to SARS-CoV-2 infection has been associated with increased plasma levels of soluble CD163.^39,40^ Nonetheless, the differential changes in macrophage phenotype and functions in response to SARS-CoV-2 infection require further investigation. Recent data suggest that SARS-CoV-2 could directly modulate macrophage signaling pathways^41^ and that such alterations could be long-lasting and might have implications for post–SARS-CoV-2 infection inflammatory syndromes.^42^

### Limitations

This study has several limitations. The study recruited only 5 patients with SARS-CoV-2 infection. The recruitment of a small cohort mainly occurred because EMB is not a routine procedure for all patients with SARS-CoV-2 infection and, consequently, previous studies examined cardiac specimens that were mostly obtained from autopsies. Furthermore, the small cohort does not allow us to assess the potential consequences of immunosuppressant drugs.

Second, the differential regulation of MAPK and B-Raf pathways was identified on the protein level but not in the transcriptome, which might be owing to a methodological limitation. When using challenging tissues, such as FFPE biopsy samples, RNA decay may compromise the analysis, whereas proteins are more stable, and their degradation is substantially less pronounced.^43^ This issue highlights the importance of mass spectrometry–based proteomic approaches when conducting FFPE analysis. Nonetheless, our analysis of the complement cascade revealed differential regulation on both the RNA and protein levels, supporting the feasibility of RNA sequencing in this setting.

Third, we did not detect SARS-CoV-2 RNA in our EMB specimens, suggesting an absence or only small amounts of virus-infected cardiac cells, whereas SARS-CoV-2 RNA was identified in cardiac autopsies of patients with infection.^3,44^ For instance, Bearse et al^45^ detected SARS-CoV-2–positive cells in the cardiac tissue of most patients with fatal SARS-CoV-2 infection. However, the density of infected cells, as analyzed by in situ hybridization, was very low. Thus, SARS-CoV-2 RNA may be missed in small tissue samples, such as those obtained from EMB. Other studies addressing SARS-CoV-2 tropism found that viral loads in tissues outside the respiratory system were low and highly variable, and that mean cardiac RNA values were within the range of the blood and the nervous systems.^46,47^

## Conclusions

In this case series, myocardial inflammation associated with SARS-CoV-2 infection was characterized by a cellular immune infiltrate that was dominated by macrophages expressing C1q and CD163. Deep phenotyping revealed substantial upregulation of MAPK-associated pathways as well as upregulation of the complement system. Although the results of this case series require confirmation in prospective studies with larger numbers of patients, the findings may open new avenues of research into the treatment of inflammatory cardiomyopathies. The present study provides proof of concept for multimodal analysis of cardiac FFPE biopsy material, which may improve the diagnosis and treatment of heart diseases in the future.^48^

## Notes

Supplement 1. **eMethods.** Tissue Preparation for Proteomics, LC-MS/MS Analysis, MS Data Processing and Analysis, RNA Analysis, and Immunofluorescence Staining and Confocal Imaging **eTable 1.** Clinical Characteristics of Patients With SARS-CoV-2 Infection **eTable 2.** Participant Demographic and Clinical Characteristics **eTable 3.** RNA Quality **eFigure 1.** Schematic for Processing and Analysis of EMB Specimens **eFigure 2.** Quality Assessment of Proteomic Data **eFigure 3.** Proteomic Comparison Between Groups **eFigure 4.** Identification of Angiotensin-Converting Enzyme 2 (ACE2) **eFigure 5.** Common Protein Significance of Inflammatory Conditions vs Noninflammatory Control Condition **eFigure 6.** Distinct Protein Significance of SARS-CoV-2 Group vs Noninflammatory Control Group **eFigure 7.** Heat Map of RNA Transcripts With Significance in Gene Set Enrichment Analysis **eFigure 8.** Proteomic Profile of Complement System **eAppendix.** Subchain Expansions Figure 3
eReferences

Supplement 2. **Nonauthor Collaborators.** The EMB Study Group

## References

[1] Tschöpe C, Ammirati E, Bozkurt B, . Myocarditis and inflammatory cardiomyopathy: current evidence and future directions. Nature Rev Cardiol. 2021;18(3):169-193. doi:10.1038/s41569-020-00435-x 33046850 PMC7548534

[2] Kühl U, Pauschinger M, Noutsias M, . High prevalence of viral genomes and multiple viral infections in the myocardium of adults with “idiopathic” left ventricular dysfunction. Circulation. 2005;111(7):887-893. doi:10.1161/01.CIR.0000155616.07901.35 15699250

[3] Lindner D, Fitzek A, Bräuninger H, . Association of cardiac infection with SARS-CoV-2 in confirmed COVID-19 autopsy cases. JAMA Cardiol. 2020;5(11):1281-1285. doi:10.1001/jamacardio.2020.3551 32730555 PMC7385672

[4] Sandoval Y, Januzzi JL Jr, Jaffe AS. Cardiac troponin for assessment of myocardial injury in COVID-19: JACC review topic of the week. J Am Coll Cardiol. 2020;76(10):1244-1258. doi:10.1016/j.jacc.2020.06.068 32652195 PMC7833921

[5] Puntmann VO, Carerj ML, Wieters I, . Outcomes of cardiovascular magnetic resonance imaging in patients recently recovered from coronavirus disease 2019 (COVID-19). JAMA Cardiol. 2020;5(11):1265-1273. doi:10.1001/jamacardio.2020.3557 32730619 PMC7385689

[6] Caforio ALP, Pankuweit S, Arbustini E, ; European Society of Cardiology Working Group on Myocardial and Pericardial Diseases. Current state of knowledge on aetiology, diagnosis, management, and therapy of myocarditis: a position statement of the European Society of Cardiology Working Group on Myocardial and Pericardial Diseases. Eur Heart J. 2013;34(33):2636-2648, 2648a-2648d. doi:10.1093/eurheartj/eht210 23824828

[7] Cieslik M, Chugh R, Wu YM, . The use of exome capture RNA-seq for highly degraded RNA with application to clinical cancer sequencing. Genome Res. 2015;25(9):1372-1381. doi:10.1101/gr.189621.115 26253700 PMC4561495

[8] Newton Y, Sedgewick AJ, Cisneros L, . Large scale, robust, and accurate whole transcriptome profiling from clinical formalin-fixed paraffin-embedded samples. Sci Rep. 2020;10(1):17597. doi:10.1038/s41598-020-74483-1 33077815 PMC7572424

[9] Coscia F, Doll S, Bech JM, . A streamlined mass spectrometry–based proteomics workflow for large-scale FFPE tissue analysis. J Pathol. 2020;251(1):100-112. doi:10.1002/path.5420 32154592

[10] World Medical Association. World Medical Association Declaration of Helsinki: ethical principles for medical research involving human subjects. JAMA. 2013;310(20):2191-2194. doi:10.1001/jama.2013.281053 24141714

[11] Kempen JH. Appropriate use and reporting of uncontrolled case series in the medical literature. Am J Ophthalmol. 2011;151(1):7-10.e1. doi:10.1016/j.ajo.2010.08.047 21163373 PMC3052978

[12] Meier F, Brunner AD, Frank M, . diaPASEF: parallel accumulation-serial fragmentation combined with data-independent acquisition. Nat Methods. 2020;17(12):1229-1236. doi:10.1038/s41592-020-00998-0 33257825

[13] Meier F, Brunner AD, Koch S, . Online parallel accumulation-serial fragmentation (PASEF) with a novel trapped ion mobility mass spectrometer. Mol Cell Proteomics. 2018;17(12):2534-2545. doi:10.1074/mcp.TIR118.000900 30385480 PMC6283298

[14] Dobin A, Davis CA, Schlesinger F, . STAR: ultrafast universal RNA-seq aligner. Bioinformatics. 2013;29(1):15-21. doi:10.1093/bioinformatics/bts635 23104886 PMC3530905

[15] Chetty R, Gatter K. CD3: structure, function, and role of immunostaining in clinical practice. J Pathol. 1994;173(4):303-307. doi:10.1002/path.1711730404 7525907

[16] Jiang Z, Shih DM, Xia YR, . Structure, organization, and chromosomal mapping of the gene encoding macrosialin, a macrophage-restricted protein. Genomics. 1998;50(2):199-205. doi:10.1006/geno.1998.5327 9653646

[17] Li B, Dewey CN. RSEM: accurate transcript quantification from RNA-Seq data with or without a reference genome. BMC Bioinformatics. 2011;12:323. doi:10.1186/1471-2105-12-323 21816040 PMC3163565

[18] Jassal B, Matthews L, Viteri G, . The reactome pathway knowledgebase. Nucleic Acids Res. 2020;48(D1):D498-D503. 31691815 10.1093/nar/gkz1031 PMC7145712

[19] *WebGestalt Web-Based Gene Set Analysis Toolkit*; 2019. Accessed November 10, 2021. webgestalt.org

[20] Carvelli J, Demaria O, Vély F, ; Explore COVID-19 IPH Group; Explore COVID-19 Marseille Immunopole Group. Association of COVID-19 inflammation with activation of the C5a-C5aR1 axis. Nature. 2020;588(7836):146-150. doi:10.1038/s41586-020-2600-6 32726800 PMC7116884

[21] Hoffmann M, Kleine-Weber H, Schroeder S, . SARS-CoV-2 cell entry depends on ACE2 and TMPRSS2 and is blocked by a clinically proven protease inhibitor. Cell. 2020;181(2):271-280. doi:10.1016/j.cell.2020.02.052 32142651 PMC7102627

[22] De Silva AM, Gallardo A, Fraser DA. Macrophage production and activity of innate immune proteins C1q, C1r, and C1s are modulated in response to molecular patterns. J Immunol. 2020;204(1)(suppl):226.

[23] Kyriakis JM, Avruch J. Mammalian MAPK signal transduction pathways activated by stress and inflammation: a 10-year update. Physiol Rev. 2012;92(2):689-737. doi:10.1152/physrev.00028.2011 22535895

[24] Rose BA, Force T, Wang Y. Mitogen-activated protein kinase signaling in the heart: angels versus demons in a heart-breaking tale. Physiol Rev. 2010;90(4):1507-1546. doi:10.1152/physrev.00054.2009 20959622 PMC3808831

[25] Ai X, Yan J, Carrillo E, Ding W. The stress-response MAP kinase signaling in cardiac arrhythmias. Rev Physiol Biochem Pharmacol. 2016;172:77-100. doi:10.1007/112_2016_8 27848025 PMC6791713

[26] Correll RN, Goonasekera SA, van Berlo JH, . STIM1 elevation in the heart results in aberrant Ca^2+^ handling and cardiomyopathy. J Mol Cell Cardiol. 2015;87:38-47. doi:10.1016/j.yjmcc.2015.07.032 26241845 PMC4637225

[27] Lo MW, Kemper C, Woodruff TM. COVID-19: complement, coagulation, and collateral damage. J Immunol. 2020;205(6):1488-1495. doi:10.4049/jimmunol.2000644 32699160 PMC7484432

[28] Annane D, Heming N, Grimaldi-Bensouda L, ; Garches COVID 19 Collaborative Group. Eculizumab as an emergency treatment for adult patients with severe COVID-19 in the intensive care unit: a proof-of-concept study. EClinicalMedicine. 2020;28:100590. doi:10.1016/j.eclinm.2020.100590 33173853 PMC7644240

[29] Yan B, Freiwald T, Chauss D, . SARS-CoV-2 drives JAK1/2-dependent local complement hyperactivation. Sci Immunol. 2021;6(58):eabg0833. doi:10.1126/sciimmunol.abg0833 33827897 PMC8139422

[30] Gutmann C, Takov K, Burnap SA, . SARS-CoV-2 RNAemia and proteomic trajectories inform prognostication in COVID-19 patients admitted to intensive care. Nat Commun. 2021;12(1):3406. doi:10.1038/s41467-021-23494-1 34099652 PMC8184784

[31] Nicholson-Weller A, Wang CE. Structure and function of decay accelerating factor CD55. J Lab Clin Med. 1994;123(4):485-491. 7511675

[32] Cicardi M, Zingale L, Zanichelli A, Pappalardo E, Cicardi B. C1 inhibitor: molecular and clinical aspects. Springer Semin Immunopathol. 2005;27(3):286-298. doi:10.1007/s00281-005-0001-4 16267649

[33] Ramlall V, Thangaraj PM, Meydan C, . Immune complement and coagulation dysfunction in adverse outcomes of SARS-CoV-2 infection. Nat Med. 2020;26(10):1609-1615. doi:10.1038/s41591-020-1021-2 32747830 PMC7809634

[34] Diao B, Wang C, Wang R, . Human kidney is a target for novel severe acute respiratory syndrome coronavirus 2 (SARS-CoV-2) infection. medRxiv. Preprint posted online April 10, 2020. doi:10.1101/2020.03.04.20031120

[35] Lubbers R, van Essen MF, van Kooten C, Trouw LA. Production of complement components by cells of the immune system. Clin Exp Immunol. 2017;188(2):183-194. doi:10.1111/cei.12952 28249350 PMC5383442

[36] Son M, Porat A, He M, . C1q and HMGB1 reciprocally regulate human macrophage polarization. Blood. 2016;128(18):2218-2228. doi:10.1182/blood-2016-05-719757 27683415 PMC5095756

[37] Fabriek BO, van Bruggen R, Deng DM, . The macrophage scavenger receptor CD163 functions as an innate immune sensor for bacteria. Blood. 2009;113(4):887-892. doi:10.1182/blood-2008-07-167064 18849484

[38] Bajpai G, Schneider C, Wong N, . The human heart contains distinct macrophage subsets with divergent origins and functions. Nat Med. 2018;24(8):1234-1245. doi:10.1038/s41591-018-0059-x 29892064 PMC6082687

[39] Gómez-Rial J, Currás-Tuala MJ, Rivero-Calle I, . Increased serum levels of sCD14 and sCD163 indicate a preponderant role for monocytes in COVID-19 immunopathology. Front Immunol. 2020;11:560381. doi:10.3389/fimmu.2020.560381 33072099 PMC7538662

[40] Zingaropoli MA, Nijhawan P, Carraro A, . Increased sCD163 and sCD14 plasmatic levels and depletion of peripheral blood pro-inflammatory monocytes, myeloid and plasmacytoid dendritic cells in patients with severe COVID-19 pneumonia. Front Immunol. 2021;12:627548. doi:10.3389/fimmu.2021.627548 33777012 PMC7993197

[41] Abdelmoaty M, Yeapuri P, Machhi J, . Defining the immune responses for SARS-CoV-2-human macrophage interactions. bioRxiv. Preprint posted online July 15, 2021. 10.3389/fimmu.2021.741502 PMC8521106 34671355

[42] Theobald SJ, Simonis A, Georgomanolis T, . Long-lived macrophage reprogramming drives spike protein–mediated inflammasome activation in COVID-19. EMBO Mol Med. 2021;13(8):e14150. doi:10.15252/emmm.202114150 34133077 PMC8350892

[43] Shao W, Guo T, Toussaint NC, . Comparative analysis of mRNA and protein degradation in prostate tissues indicates high stability of proteins. Nat Commun. 2019;10(1):2524. doi:10.1038/s41467-019-10513-5 31175306 PMC6555818

[44] Delorey TM, Ziegler CGK, Heimberg G, . COVID-19 tissue atlases reveal SARS-CoV-2 pathology and cellular targets. Nature. 2021;595(7865):107-113. doi:10.1038/s41586-021-03570-8 33915569 PMC8919505

[45] Bearse M, Hung YP, Krauson AJ, . Factors associated with myocardial SARS-CoV-2 infection, myocarditis, and cardiac inflammation in patients with COVID-19. *Mod Pathol*. 2021;34(7):1345-1357. 10.1038/s41379-021-00790-1 PMC9813560 33727695

[46] Puelles VG, Lütgehetmann M, Lindenmeyer MT, . Multiorgan and renal tropism of SARS-CoV-2. N Engl J Med. 2020;383(6):590-592. doi:10.1056/NEJMc2011400 32402155 PMC7240771

[47] Deinhardt-Emmer S, Wittschieber D, Sanft J, . Early postmortem mapping of SARS-CoV-2 RNA in patients with COVID-19 and the correlation with tissue damage. Elife. 2021;10:e60361. doi:10.7554/eLife.60361 33781385 PMC8009677

[48] Seferović PM, Tsutsui H, McNamara DM, . HFA/HFSA/JHFS position statement on endomyocardial biopsy. J Card Fail. Published online May 2021. 10.1016/j.cardfail.2021.04.010 34022400

