# Supplementary material for: Association of Complement and MAPK Activation With SARS-CoV-2–Associated Myocardial Inflammation
Source: JAMA Cardiol. 2021 Dec 15;7(3):286–97. doi: 10.1001/jamacardio.2021.5133 (PMC8674808; doi:10.1001/jamacardio.2021.5133)
Supplement: Supplement 1. — eMethods. Tissue Preparation for Proteomics, LC-MS/MS Analysis, MS Data Processing and Analysis, RNA Analysis, and Immunofluorescence Staining and Confocal Imaging eTable 1. Clinical Characteristics of Patients With SARS-CoV-2 Infection eTable 2. Participant Demographic and Clinical Characteristics eTable 3. RNA Quality eFigure 1. Schematic for Processing and Analysis of EMB Specimens eFigure 2. Quality Assessment of Proteomic Data eFigure 3. Proteomic Comparison Between Groups eFigure 4. Identification of Angiotensin-Converting Enzyme 2 (ACE2) eFigure 5. Common Protein Significance of Inflammatory Conditions vs Noninflammatory Control Condition eFigure 6. Distinct Protein Significance of SARS-CoV-2 Group vs Noninflammatory Control Group eFigure 7. Heat Map of RNA Transcripts With Significance in Gene Set Enrichment Analysis eFigure 8. Proteomic Profile of Complement System eAppendix. Subchain Expansions Figure 3 eReferences [file jamacardiol-e215133-s001.pdf]

Supplemental Online Content

Weckbach LT, Schweizer L, Kraechan A, et al; EMB Study Group. Association of complement and MAPK activation with SARS-CoV-2–associated myocardial inflammation. *JAMA Cardiol*. Published online December 15, 2021. doi:10.1001/jamacardio.2021.5133

eMethods. Tissue Preparation for Proteomics, LC-MS/MS Analysis, MS Data Processing and Analysis, RNA Analysis, and Immunofluorescence Staining and Confocal Imaging

eTable 1. RNA Quality

eTable 2. Clinical Characteristics of Patients With SARS-CoV-2 Infection

eTable 3. Participant Demographic and Clinical Characteristics

eFigure 1. Schematic for Processing and Analysis of EMB Specimens

eFigure 2. Quality Assessment of Proteomic Data

eFigure 3. Proteomic Comparison Between Groups

eFigure 4. Identification of Angiotensin-Converting Enzyme 2 (ACE2)

eFigure 5. Common Protein Significance of Inflammatory Conditions vs Noninflammatory Control Condition

eFigure 6. Distinct Protein Significance of SARS-CoV-2 Group vs Noninflammatory Control Group

eFigure 7. Heat Map of RNA Transcripts With Significance in Gene Set Enrichment Analysis

eFigure 8. Proteomic Profile of Complement System

eAppendix Subchain Expansions Figure 3

eReferences

This supplementary material has been provided by the authors to give readers additional information about their work.

© 2021 Weckbach LT et al. *JAMA Cardiology*.

eMethods. Tissue Preparation for Proteomics, LC-MS/MS Analysis, MS Data Processing and Analysis, RNA Analysis, and Immunofluorescence Staining and Confocal Imaging

Tissue preparation for proteomics

FFPE material was collected from EMB sections (3 replicates, 5 µm) and processed in a 96-well format based on computer-generated randomization patterns. Subsequently, sample lysis and thorough deparaffinization were accomplished using a hybrid of established protocols and focused sonification [2,3]. In brief, samples were supplemented by 40 µL of tissue lysis buffer (truXTRAC Proteins - Tissue Lysis Buffer, Prod. No. 520284, Covaris), heating at 90°C and focused sonification (LE220-plus, Covaris; peak Power: 450.0, duty factor: 50%, cycles: 200, average power: 225, time: 300 sec). Next, proteins were reduced, alkylated and aggregated on magnetic carboxylate modified particles (Sera-Mag™, Prod. No. 24152105050350, GE Healthcare/ Merck KGaA, Darmstadt, Germany). For paraffin removal, beads were washed conducting three consecutive steps in 100% isopropanol for 10 min at 50°C and 1400 rpm. Beads were then resuspended in 100 µL of 100 mM Tris, pH 8.5, and supplemented with 0.5 µg trypsin and LysC for an overnight digest at 37°C and 1300 rpm. Subsequently, protein digestion was completed by the addition of 0.25 µg trypsin and LysC, respectively, and incubation was resumed for four hours. Resulting supernatant was collected, the enzymatic reaction was quenched using TFA at a final concentration of 1% (v/v) and peptides were purified using two-layer SDB-RPS (Empore™ SPE Disks, CDS Analytical, 98-0604-0226-4) StageTips. Purified samples were vacuum-dried and resuspended in injection buffer (2% acetonitrile, 0.1% TFA in H2O). To generate group-specific reference libraries, 50 µg of peptides were pooled for each sample type and pre-fractionated into 8 fractions by high-pH reversed-phase chromatography as described earlier [4].

LC-MS/MS analysis

Liquid chromatography (LC)-mass spectrometry (MS) analysis was performed on an EASY-nLC 1200 ultrahigh-pressure system (Thermo Fisher Scientific, San Jose, USA) coupled to a hybrid TIMS quadrupole TOF mass spectrometer (timsTOF Pro, Bruker, Billerica, USA) via a CaptiveSpray nano-electrospray ion source. For each sample, 200 ng of peptides were separated on a 50cm-column (inner diameter: 75µm, generated in-house using ReproSil-Pur C18-AQ 1.9µm beads [Dr. Maisch GmbH]) and a total gradient length of 120 min. While the oven temperature was kept at constant 60°C, elution was performed on a gradient of buffer A (0.1% FA in H20) and buffer B (80% acetonitrile, 0.1 % FA in H20). Herein, buffer B was increased from 3% to 30% with 95 min, followed by an additional step to 60 % within 5 min and a final plateau of 95% for 5 min to ensure peptide elution. A constant flow rate 300 nl/min was kept for all measurements. MS data were recorded in duplicates for each sample using the standard method of data-independent acquisition embedded in the novel parallel accumulation–serial fragmentation method (diaPASEF) as introduced recently [5]. Fractions of each peptide library were measured in a top 10 data-dependent acquisition (DDA) PASEF mode [6]. In detail, for each acquisition cycle, one TIMS-MS scan was followed by 10 PASEF MS/MS scans (precursor isolation window: 2 Th for m/z < 700, 3 Th for m/z >700, isolation range: 100-1,700 m/z; dynamic exclusion: 24 s). Ion mobility values were covered from 1/K0 = 1.6 Vs cm-2 to 0.6 Vs cm-2 using ramp times of 100 ms in the TIMS analyzer. For MS2 scans of both acquisition modes, collision energies were defined linearly to the ion mobility from 59 eV at 1/K0=1.6Vs cm−2 to 20 eV at 1/K0=0.6Vs cm−2. Precursor ions of single charges were excluded using a polygon filter. MS data acquisition was performed on the otof control software (version 6.2, Bruker Daltonik GmbH).

MS data processing and analysis

Data were processed in Spectronaut version 14.9.201124.47784 (Biognosys AG, Schlieren, Switzerland) using the databases for human (software-integrated) and for severe acute respiratory syndrome coronavirus 2 (Uniprot, downloaded on February 16 2020, Swiss-Prot) using standard settings. Spectral libraries were generated from the DDA data of the pre-fractionated samples pools using the Pulsar Search engine separately for each group. Subsequently, DIA data of each sample were associated to the respective spectral library and jointly quantified. Bioinformatics analyses of all MS data were performed using the R statistical computing environment version 4.0.2. In preparation for the analysis, protein intensities were log2-transformed and the mean value of the technical replicates was calculated for each protein where possible. Next, we filtered stringently for 60% valid intensity

© 2021 Weckbach LT et al. *JAMA Cardiology*.

values present in each of the four patient groups. For the pairwise comparisons between groups, fold changes were determined with the omission of NA values and a statistical t-test was performed. Based on the resulting p-values, q-values of less than 0.01 and a minimal fold change of 2 were considered to be significant. Overrepresentation analysis (ORA) was accomplished using the WebGestalt gene set analysis toolkit (version 0.4.4) in reference to the Reactome Pathway Database and Benjamini-Hochberg FDR. Pearson correlation values of the proteome were calculated based on the mean abundances of each protein within the respective sample group. Upset plots were generated using the Package UpSetR (version 1.4.0). Further plots were assembled using the ggplot2 (version 3.3.3) and ComplexHeatmap (version 2.4.3) packages. For a Principle Component Analysis (PCA), missing intensity values were imputed sample-wise based on a normal distribution (width of 0.3, downshift of 1.8) using the FactoMineR package (version 2.4).

RNA analysis

RNA isolation of 19 EMB FFPE samples was carried out with the AllPrep DNA/RNA Mini Kit from Qiagen (Prod. No. 80284) according to the manufacturer's protocol. RNA yield and DV200 values, which indicate the percentage of RNA fragments with a nucleotide size larger than 200, are provided in eTable 3. Sequencing libraries were generated with the Illumina TruSeq® RNA Exome technology. After the limited cycle PCR at the end of the library preparation all samples were quality controlled. Therefore, the DNA 1000 and High Sensitivity DNA LabChip kit was used on the 2100 Bioanalyzer (Agilent Technologies). Furthermore, all libraries were quantified using the highly sensitive fluorescent dye-basedQubit®ds DNA HS Assay kit (Thermo Fisher Scientific). RNA sequencing was performed on the Illumina NovaSeq™ 6000 next generation sequencing system (2 x 75 bp PE run). Sequencing reads were aligned to the human reference genome (version GRCH38.100) with STAR (version 2.7.3). Expression values (TPM) were calculated with RSEM (version 1.3.3). Differential gene expression analysis was performed using DEseq2 (version 1.28.1). An adjusted p value (FDR) of less than 0.1 was set to classify significantly changed expression. Pathway enrichment analysis was performed using ReactomePA (version 1.34.0).

Immunofluorescence staining and confocal imaging

Images were acquired with a LSM 880 (Zeiss) confocal microscope with Airyscan module. The imaging parameters were as follows: image size (166x166 µm), z stack range (19µm), objective Plan-Apochromat 20x/0.8, laser intensity 488 nm (1.0%), 514 nm (1.0%) and 405 nm (1.0%). Images were analyzed with ZEN software using the histogram plug-in. A defined intracellular region of interest (ROI) of 1 μm^2 was applied to obtain the median fluorescence intensity (MFI) from each cytoplasmic cell region. Imaging parameters for excitation, detection and all software settings were identical for all imaged samples, allowing calculation and comparison of MFI values for indicated proteins (C1q, CD163). Results are indicated as median (MFI) with interquartile range.

© 2021 Weckbach LT et al. *JAMA Cardiology*.

eTable 1. RNA Quality

Samples	RNA total amount (ng)	DV_{200}
SARS-CoV-2	20.54	63
SARS-CoV-2	49.4	63
SARS-CoV-2	17.55	63
SARS-CoV-2	6.24	55
SARS-CoV-2	23.40	75
Non-inflammatory control	85.15	65
Non-inflammatory control	67.08	66
Non-inflammatory control	119.60	66
Non-inflammatory control	39.00	63
Non-inflammatory control	15.86	54
Virus-associated myocarditis	51.74	51
Virus-associated myocarditis	58.63	56
Virus-associated myocarditis	60.32	52
Virus-associated myocarditis	51.48	60
Immune-mediated myocarditis	36.14	50
Immune-mediated myocarditis	24.18	48
Immune-mediated myocarditis	31.72	52
Immune-mediated myocarditis	75.40	58
Immune-mediated myocarditis	72.80	61

© 2021 Weckbach LT et al. *JAMA Cardiology*.

eTable 2. Clinical Characteristics of Patients With SARS-CoV-2 Infection

	SARS-CoV-2[a]				
**Sex**	m	f	m	m	m
**Symptom at admission**	fever	confusion	fever	fever	dyspnea
**TnT (ng/ml)[b]**	0.025	0.135	0.587	0.067	0.015
**NT-proBNP (pg/ml)[b]**	387	9348	536	92	1138
**CRP (mg/dl)[b]**	2.5	1.2	2.8	2.8	1.1
**IL-6 (pg/ml)[b]**	34	47	34	14	4
**LV-GLS (Echo)**	-13.9	-5.3	-9.6	-8.4	-3.5
**Myocardial tissue injury[c] (MRI)**	yes	yes	yes	yes	yes
**Distribution of myocardial tissue injury (MRI)**	focal	focal	global	global	patchy
**Edema (MRI)**	yes	yes	yes	no	no
**Pericardial effusion (MRI)**	yes	yes	no	yes	no
**Lake Louise criteria[d] met**	yes	yes	yes	no	no
**Discharge from hospital**	yes	yes	yes	yes	yes

ICU, intensive care unit; TnT, troponin T; NT-proBNP, N-terminal pro brain natriuretic peptide; CRP, c-reactive protein; IL-6, interleukin 6; LV-GLS, left ventricular global longitudinal strain
[a] Age range: 41-76 years
[b] At time of biopsy
[c] Myocardial tissue injury was defined by presence of late gadolinium enhancement, elevated extracellular volume or prolonged native T1 relaxation time [1]
[d] Main criteria: Non-ischemic myocardial injury + myocardial edema [1]

© 2021 Weckbach LT et al. *JAMA Cardiology*.

eTable 3. Participant Demographic and Clinical Characteristics

	SARS-CoV-2					Non-inflammatory control					Virus-associated myocarditis				Immune-mediated myocarditis				
**Sex**	m	m	f	m	m	f	m	m	m	m	m	m	m	m	m	f	m	f	m
**CVRF, no.**	≤1	≤1	≤1	>1	≤1	≤1	>1	≤1	≤1	≤1	≤1	≤1	≤1	≤1	>1	≤1	≤1	≤1	≤1
**CAD**	no	no	no	no	no	?	no	no	no	no	no	no	?	no	no	yes	no	no	no
**NYHA**	III	III	IV	IV	III	III	III	II	II	II	III	IV	III	III	III	III	II	?	IV
**Arrhythmia**[a]	no	no	no	no	no	no	no	no	yes	no	no	no	no	no	yes	no	no	no	no
**LVEF (%)**	40-55	>55	30-39	>55	>55	30-39	<30	40-55	?	< 30	<30	<30	>55	30–39	30–39	< 30	40–55	<30	<30
**ICU**[b]	no	no	yes	yes	no	no	no	no	?	no	no	yes	no	no	no	?	no	yes	no
**Immunosuppression**	no	yes	no	yes	no	no	no	no	no	no	no	no	no	no	no	no	no	no	no

CVRF, cardiovascular risk factors; CAD, coronary artery disease; ICU, intensive care unit; TnT, troponin T; NYHA, New York Heart Association class; LVEF, left ventricular ejection fraction; ICU, intensive care unit

[a] includes sustained and non-sustained ventricular tachycardia

[b] at time of biopsy

© 2021 Weckbach LT et al. *JAMA Cardiology*.

eFigure 1. Schematic for Processing and Analysis of EMB Specimens

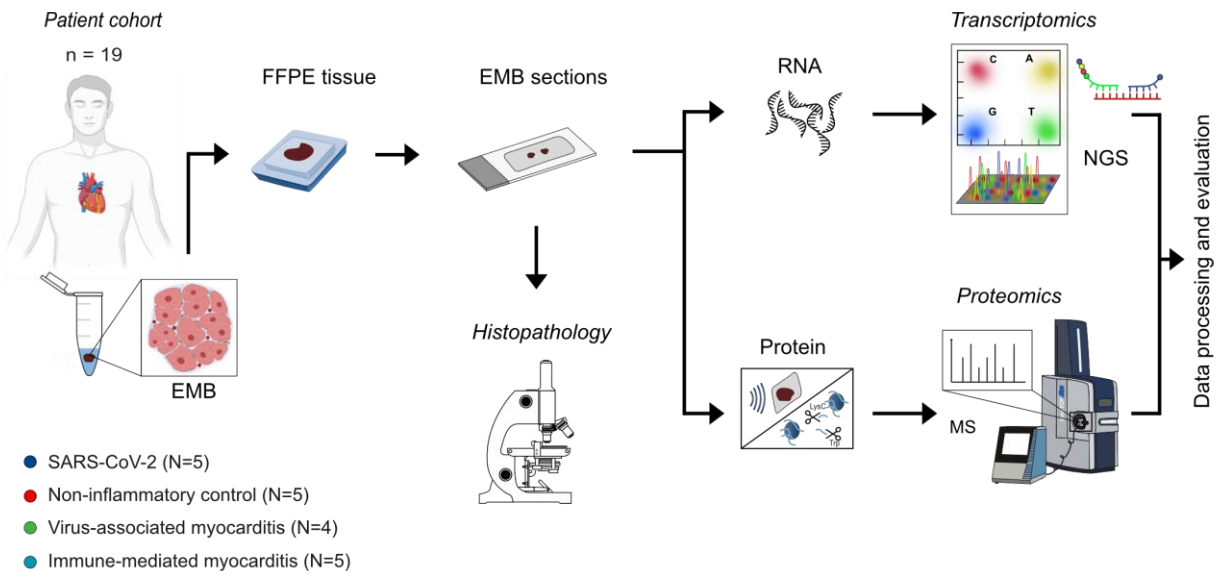

A total of 19 patients were analysed in this study. Subsequent to EMB and paraffin embedding, samples were characterized by histopathology and subjected to RNA-sequencing and mass spectrometry-based proteomics.

© 2021 Weckbach LT et al. *JAMA Cardiology*.

eFigure 2. Quality Assessment of Proteomic Data

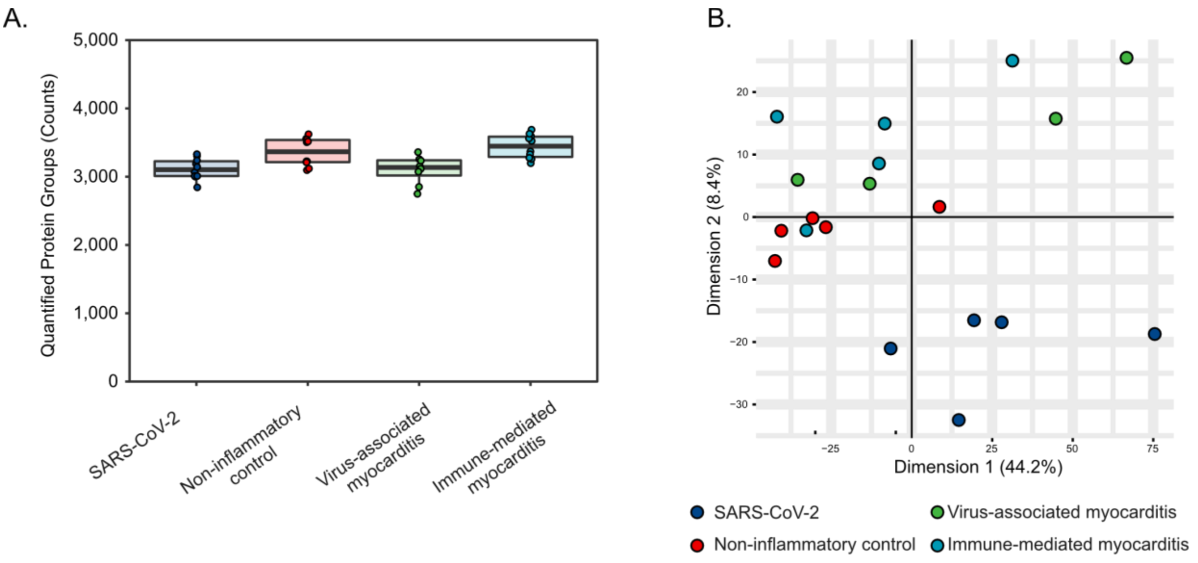

A. Count of quantified protein groups for the four groups of the EMB cohort in this study. Points display the distribution of counts within each group while each box represents the median and +/- interquartile range of these points. B. Principal component analysis of all four groups. The two dimensions indicate the highest variance of components. Each color represents a group of the study as indicated.

© 2021 Weckbach LT et al. *JAMA Cardiology*.

eFigure 3. Proteomic Comparison Between Groups

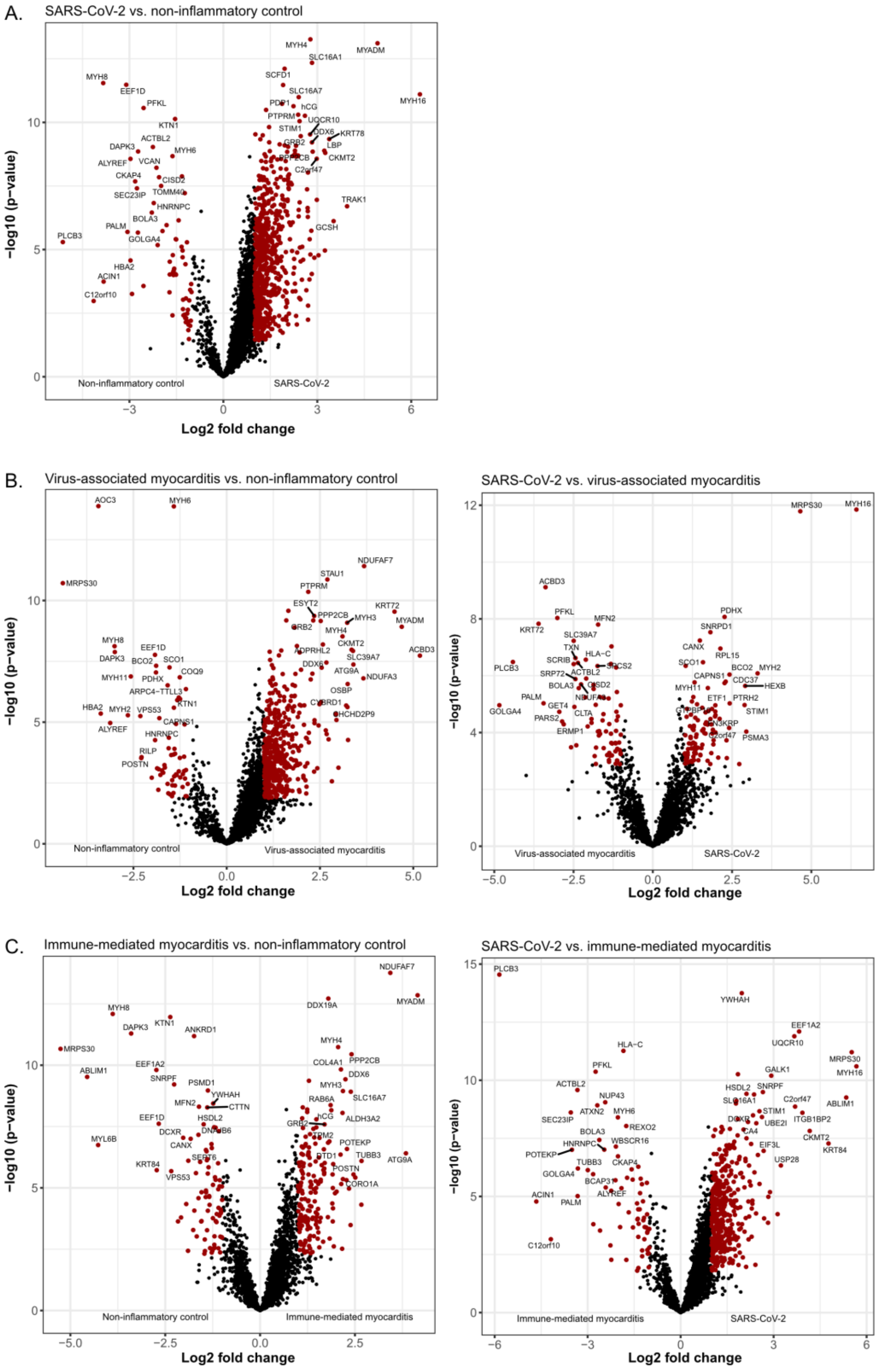

Volcano Plots of the proteomic comparison between A. SARS-CoV-2 vs. non-inflammatory controls, B. Virus-associated myocarditis vs. SARS-CoV-2 or non-inflammatory controls, respectively, and C. Immune-mediated myocarditis vs. SARS-CoV-2 or non-inflammatory controls, respectively. Significant differential expression (q-value < 0.01 and a minimum fold change of 2) of proteins is highlighted in red.

© 2021 Weckbach LT et al. *JAMA Cardiology*.

eFigure 4. Identification of Angiotensin-Converting Enzyme 2 (ACE2)

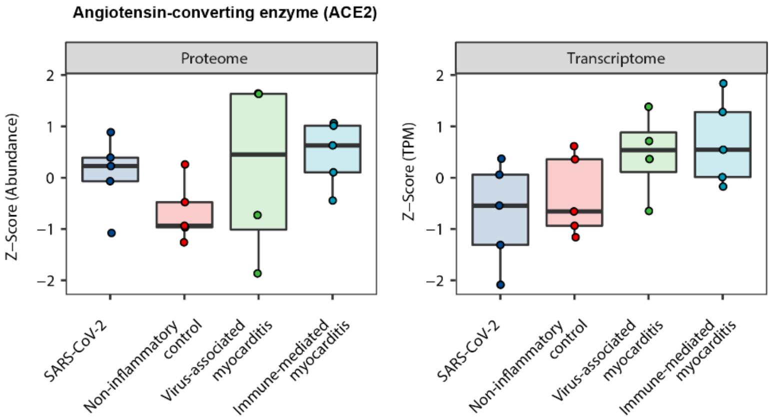

Z-Score normalized abundances and TPM values of the angiotensin-converting enzyme 2 (ACE2) on the proteomic and transcriptomic level. The difference in protein abundances was not significant (t-test, q-value < 0.01, fold change > 2) in the respective comparisons between groups. Points display the distribution of counts within each group while each box represents the median and +/- interquartile range of these points.

© 2021 Weckbach LT et al. *JAMA Cardiology*.

eFigure 5. Common Protein Significance of Inflammatory Conditions vs Noninflammatory Control Condition

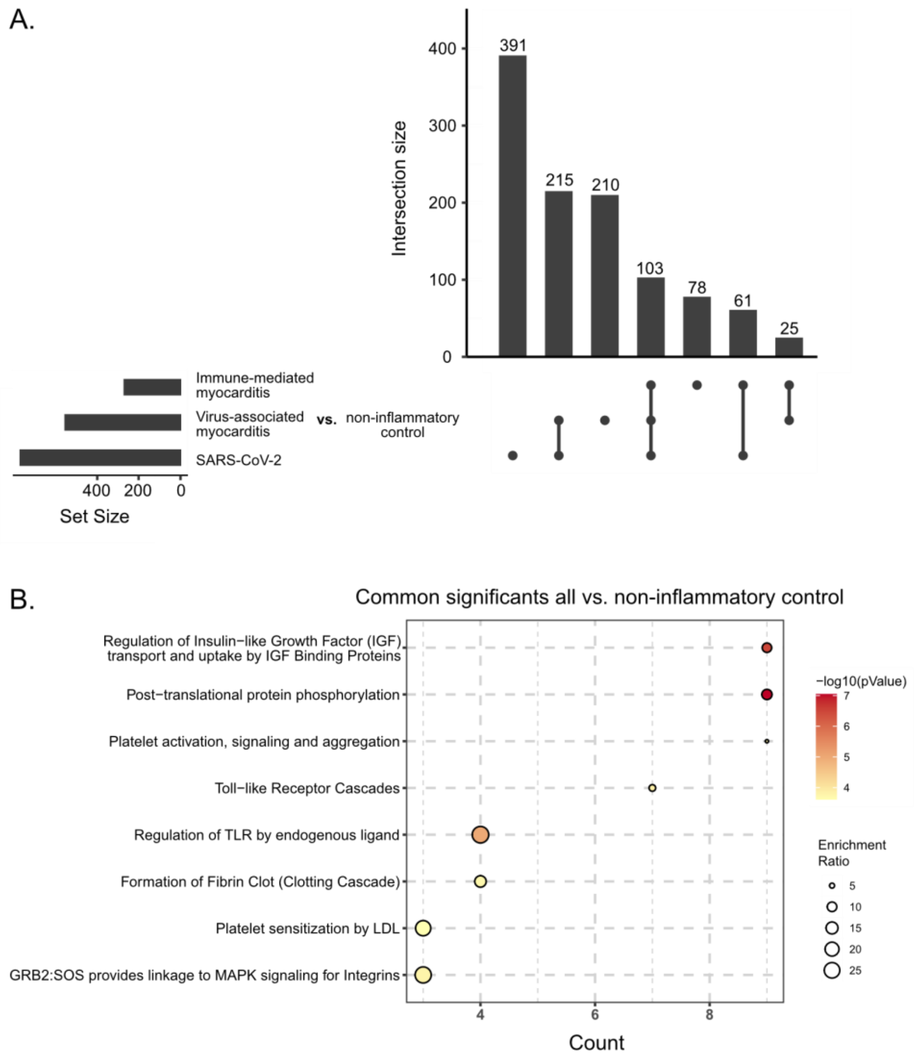

A. Shared or unique differential expression of significant proteins in the specific comparisons between SARS-CoV-2 infection, viral, and immune-mediated myocarditis to non-inflammatory controls, respectively. The number of significant proteins within each comparison is depicted by the set size, whereas the intersection size represents the overlap quantity as shown by the points below. B. Overrepresentation analysis of significant proteins (t-test, q-value < 0.01, fold change > 2) occurring jointly in the comparisons against non-inflammatory control as shown in A. Top 15 significantly enriched terms of the 'REACTOME pathway database' (FDR cutoff: 0.05, Benjamini-Hochberg correction) were ordered by decreasing numbers of proteins associated to each term (overlap). P-values (-log10) and enrichment ratios (Count/expected number of input genes that are annotated in the gene set) are visualized by color and point-size as indicated.

© 2021 Weckbach LT et al. *JAMA Cardiology*.

eFigure 6. Distinct Protein Significance of SARS-CoV-2 Group vs Noninflammatory Control Group

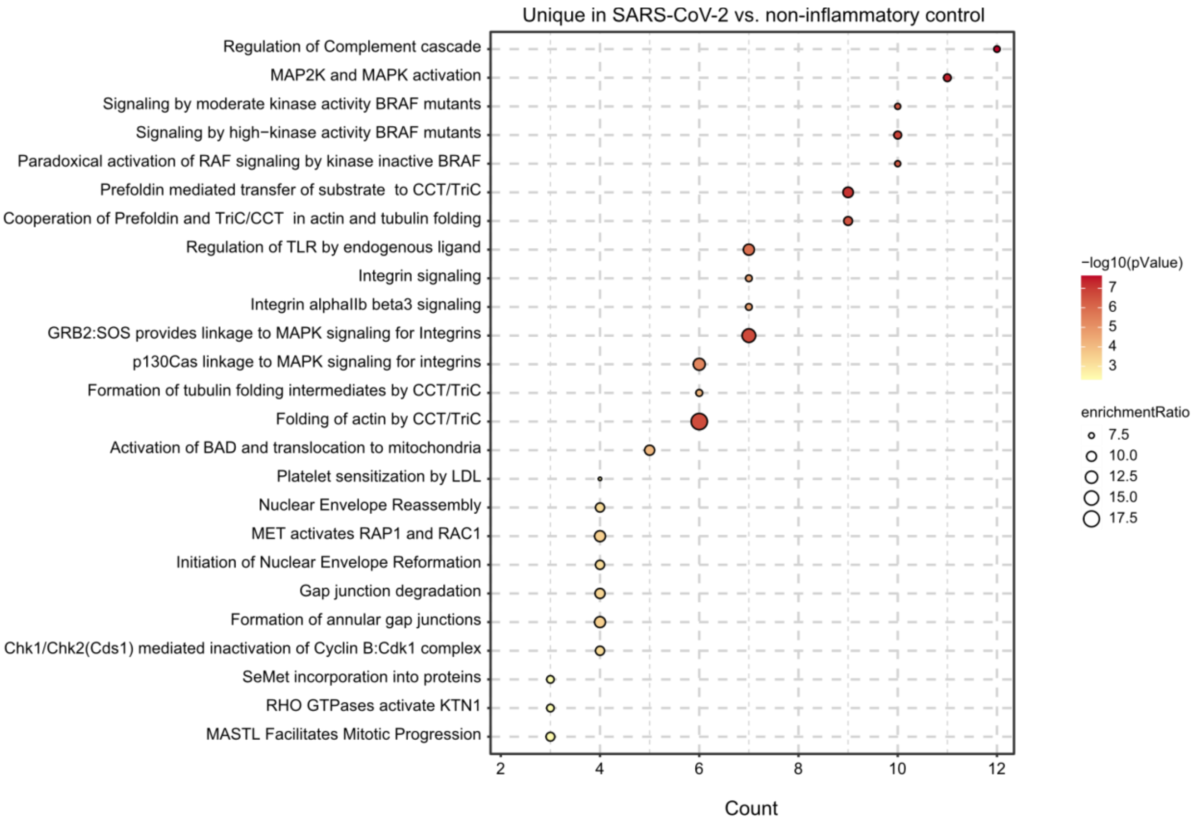

Overrepresentation analysis of significant proteins (t-test, q-value < 0.01, fold change > 2) occurring exclusively in the comparison between SARS-CoV-2 infection and non-inflammatory control of the intersection analysis in Figure 2B. Top 25 significantly enriched terms of the 'REACTOME pathway database' (FDR cutoff: 0.05, Benjamini-Hochberg correction) were ordered by decreasing numbers of proteins associated to each term (overlap). P-values (-log10) and enrichment ratios (Count/expected number of input genes that are annotated in the gene set) are visualized by color and point-size as indicated.

© 2021 Weckbach LT et al. *JAMA Cardiology*.

eFigure 7. Heat Map of RNA Transcripts With Significance in Gene Set Enrichment Analysis

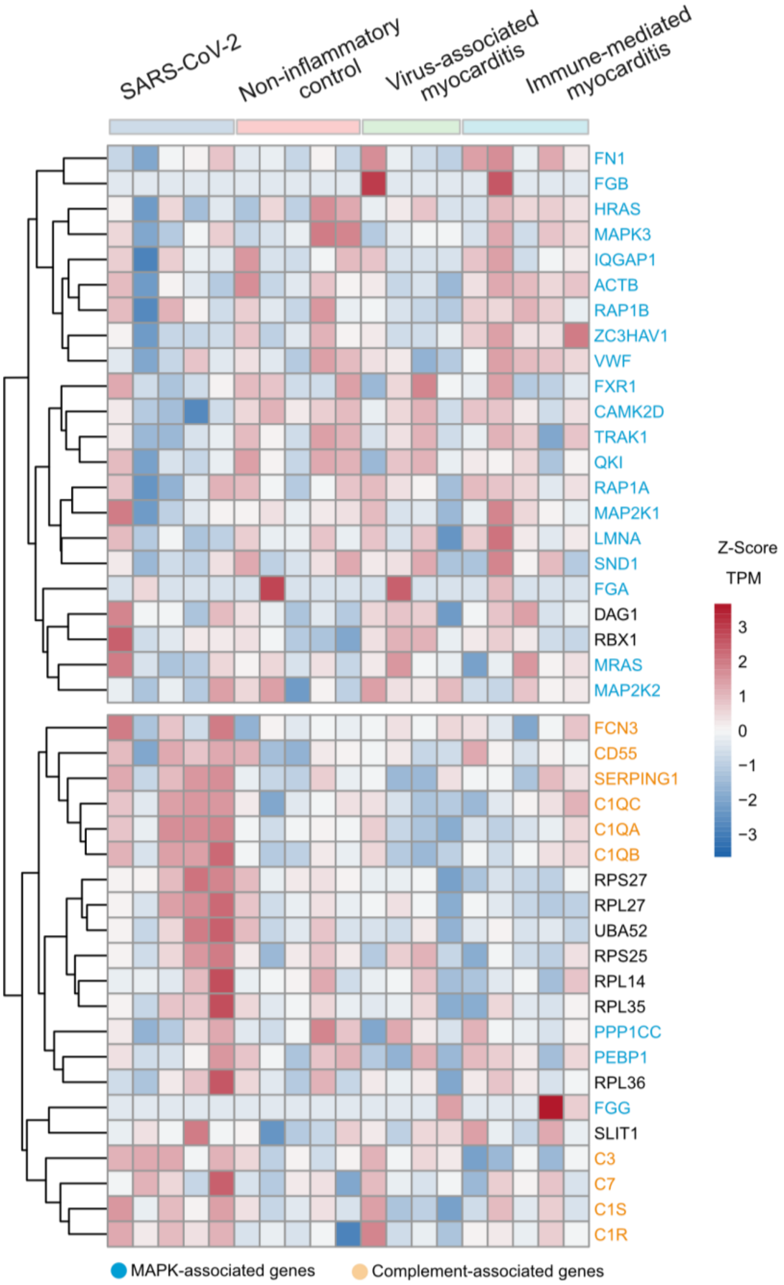

A subset of transcriptomic data was selected by significance in the gene set enrichment analysis in reference to the 'REACTOME pathway database'. Row-wise Euclidean hierarchical clustering of Z-Score TPM (Transcripts Per Million) values shows a separation into two different clusters (upper and lower). The association of each gene to MAPK signalling or the complement system is represented by color as indicated.

© 2021 Weckbach LT et al. *JAMA Cardiology*.

eFigure 8. Proteomic Profile of Complement System

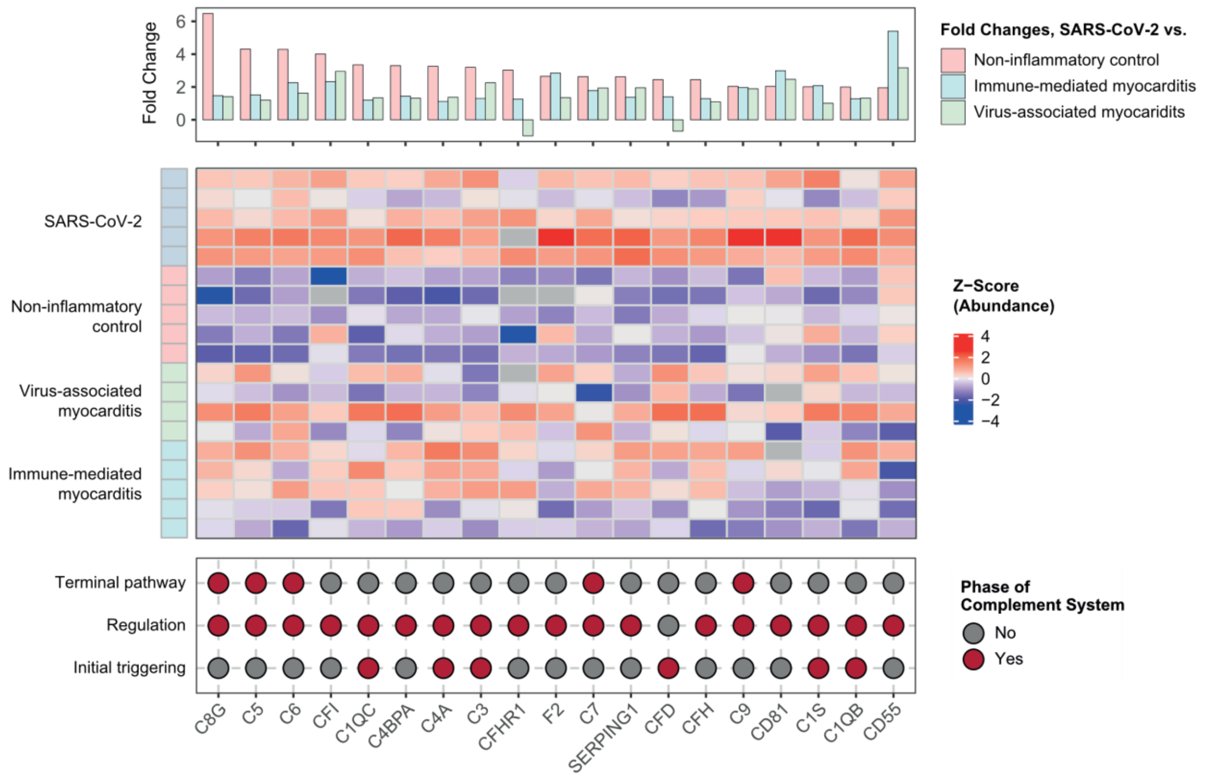

Z-Score normalized protein abundances (middle panel) of complement components which were upregulated in specimens of a SARS-CoV-2 infection compared to non-inflammatory controls in the proteomic and transcriptomic data. For each protein, the fold change between SARS-CoV-2 infection and myocarditis or controls samples (upper panel) and an annotation to the respective role in the 'REACTOME pathway database' are shown (lower panel).

© 2021 Weckbach LT et al. *JAMA Cardiology*.

eAppendix Subchain expansions Figure 3

CAMK2D-6 indicates calcium/calmodulin-dependent protein kinase type II δ chain; FGA, fibrinogen α chain; FGB, fibrinogen β chain; FGG, fibrinogen γ chain; FN1, fibronectin 1; FXR, farnesoid X receptor; HRAS, Harvey rat sarcoma virus (HRas) proto-oncogene, GTPase; IQGAP, IQ motif–containing guanosine triphosphate (GTPase) activating protein; LMNA, lamin A/C; MAP2K1/MAP2K2, mitogen-activated protein kinase 1/2; MRAS, M-Ras protein; PEBP1, phosphatidylethanolamine-binding protein 1; PPP1CC, protein phosphatase 1 catalytic subunit γ; QKI, quaking RNA-binding protein; RAP, Ras-related protein; SND, Staphylococcal nuclease domain-containing protein 1 ; TRAK1, trafficking kinesin-binding protein 1; VWF, von Willebrand factor; and ZC3HAV1, zinc finger cys–cys–cys–his (CCCH)–-type antiviral protein 1.

© 2021 Weckbach LT et al. *JAMA Cardiology*.

eReferences

1. Ferreira VM, Schulz-Menger J, Holmvang G, et al. Cardiovascular Magnetic Resonance in Nonischemic Myocardial Inflammation: Expert Recommendations. *Journal of the American College of Cardiology.* 2018;72(24):3158-3176.
2. Coscia F, Doll S, Bech JM, et al. A streamlined mass spectrometry-based proteomics workflow for large-scale FFPE tissue analysis. *The Journal of pathology.* 2020;251(1):100-112.
3. Batth TS, Tollenaere MX, Rüther P, et al. Protein Aggregation Capture on Microparticles Enables Multipurpose Proteomics Sample Preparation. *Molecular & cellular proteomics : MCP.* 2019;18(5):1027-1035.
4. Kulak NA, Geyer PE, Mann M. Loss-less Nano-fractionator for High Sensitivity, High Coverage Proteomics. *Molecular & cellular proteomics : MCP.* 2017;16(4):694-705.
5. Meier F, Brunner AD, Frank M, et al. diaPASEF: parallel accumulation-serial fragmentation combined with data-independent acquisition. *Nature methods.* 2020;17(12):1229-1236.
6. Meier F, Brunner AD, Koch S, et al. Online Parallel Accumulation-Serial Fragmentation (PASEF) with a Novel Trapped Ion Mobility Mass Spectrometer. *Molecular & cellular proteomics : MCP.* 2018;17(12):2534-2545.

© 2021 Weckbach LT et al. *JAMA Cardiology*.
