# Supplementary material for: Association of Complement and MAPK Activation With SARS-CoV-2–Associated Myocardial Inflammation
Source: JAMA Cardiol. 2021 Dec 15;7(3):286–97. doi: 10.1001/jamacardio.2021.5133 (PMC8674808; doi:10.1001/jamacardio.2021.5133)
Supplement: Supplement 2. — Nonauthor Collaborators. The EMB Study Group [file jamacardiol-e215133-s002.pdf]

Supplemental Online Content: Nonauthor Collaborators

*Indicates required information. Only first name, last name, and suffix will appear in PubMed.

*Group Name(s): EMB study group							
***First Name and Middle Initial(s)**	***Last Name**	***Suffix (eg, Jr, III)**	Academic Degrees	Institution	Location (city, state/province, country)	Role or Contribution, eg, chair, principal investigator	Group (if more than 1 Group listed in the byline) and/or Subgroup (eg, Steering Committee)
Peter	Boekstegers		MD	Helios Klinikum Siegburg	Siegburg, North Rhine-Westphalia, Germany	Co-investigator	
Thomas	Gehrig		MD	Brüderkrankenhaus Trier	Trier, Baden-Wuerttemberg, Germany	Co-investigator	
Claudius	Jacobshagen		MD	St. Vincentius-Kliniken Karlsruhe	Karlsruhe, Baden-Wuerttemberg, Germany	Co-investigator	
Werner	Moshage		MD	Klinikum Traunstein	Traunstein, Bavaria, Germany	Co-investigator	
Nikos	Werner		MD	Brüderkrankenhaus Trier	Trier, Baden-Wuerttemberg, Germany	Co-investigator	
